# Multimodal data fusion for supervised learning-based identification of USP7 inhibitors: a systematic comparison

**DOI:** 10.1186/s13321-022-00675-8

**Published:** 2023-01-11

**Authors:** Wen-feng Shen, He-wei Tang, Jia-bo Li, Xiang Li, Si Chen

**Affiliations:** 1 School of Medicine & School of Computer Engineering and Science, Shanghai University, Shanghai, 200444 China; 2 School of Pharmacy, Second Military Medical University, Shanghai, 200433 China

**Keywords:** Machine learning, Deep learning, Molecular representations, Multimodal data fusion, Ubiquitin-specific-processing protease 7

## Abstract

Ubiquitin-specific-processing protease 7 (USP7) is a promising target protein for cancer therapy, and great attention has been given to the identification of USP7 inhibitors. Traditional virtual screening methods have now been successfully applied to discover USP7 inhibitors aiming at reducing costs and speeding up time in several studies. However, due to their unsatisfactory accuracy, it is still a difficult task to develop USP7 inhibitors. In this study, multiple supervised learning classifiers were built to distinguish active USP7 inhibitors from inactive ligands. Physicochemical descriptors, MACCS keys, ECFP4 fingerprints and SMILES were first calculated to represent the compounds in our in-house dataset. Two deep learning (DL) models and nine classical machine learning (ML) models were then constructed based on different combinations of the above molecular representations under three activity cutoff values, and a total of 15 groups of experiments (75 experiments) were implemented. The performance of the models in these experiments was evaluated, compared and discussed using a variety of metrics. The optimal models are ensemble learning models when the dataset is balanced or severely imbalanced, and SMILES-based DL performs the best when the dataset is slightly imbalanced. Meanwhile, multimodal data fusion in some cases can improve the performance of ML and DL models. In addition, SMOTE, unbiased decoy selection and SMILES enumeration can improve the performance of ML and DL models when the dataset is severely imbalanced, and SMOTE works the best. Our study established highly accurate supervised learning classification models, which would accelerate the development of USP7 inhibitors. Some guidance was also provided for drug researchers in selecting supervised models and molecular representations as well as handling imbalanced datasets.

**Graphical Abstract:**

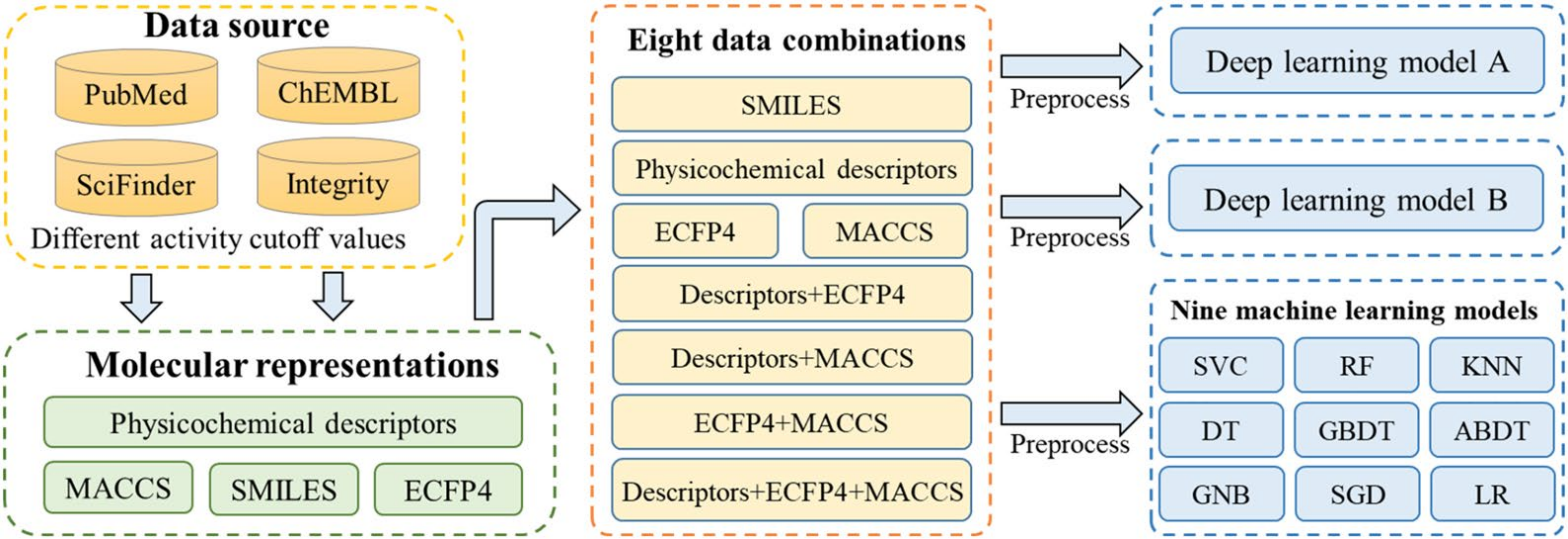

**Supplementary Information:**

The online version contains supplementary material available at 10.1186/s13321-022-00675-8.

## Introduction

Ubiquitin-specific-processing protease 7 (USP7) is a deubiquitination enzyme with the main function to release ubiquitin from the substrate protein to prevent it from being degraded [1]. Studies have identified many tumorigenesis-related proteins as substrate proteins of USP7 [2]. Thus, USP7 has been developed as a potential target for a variety of malignancies, including epithelial, prostate, breast, lung and cervical cancers [2, 3]. To date, more than 2000 USP7 small molecule inhibitors have been reported in the literature and patents. However, until now, no published USP7 inhibitors have entered clinical trials [2]. Therefore, there is a need to continue research on the discovery of USP7 inhibitors.

Drug discovery is a high-risk and high-commitment task, and traditional virtual screening methods hold the promise of accelerating drug discovery and reducing research and development costs [4]. Virtual screening can be divided into two categories, structure-based and ligand-based, depending on whether the three-dimensional structure of the target is known. These methods have now been successfully applied to discover USP7 inhibitors aiming at reducing costs and speeding up time in several studies [5–8]. However, the accuracy of the traditional virtual screening methods applied in these studies are not satisfactory and need to be further improved.

The emergence of machine learning (ML) has provided the possibility to develop drug screening models with higher accuracy [9]. With powerful learning and generalization capabilities, ML has been successfully applied to several aspects of the virtual screening pipeline, especially ligand-based virtual screening [9]. For example, Shi et al. developed a plain Bayesian model for classifying activators and non-activators of the pregnane X receptor, which was 85% accurate on the test set [10]. Deep learning (DL) is an ML method based on deep neural networks. Numerous studies have shown that models built with DL methods outperform traditional ML methods in ligand-based virtual screening, and it has even been claimed that the predictive performance of DL methods is in many cases comparable to tests performed in wet laboratories [9, 11]. The main advantage of DL methods over traditional ML methods is that their performance grows as the size of the training dataset increases. DL algorithms do not perform well when there is very little data because they need a large amount of data to understand it perfectly. However, traditional ML algorithms perform better when the amount of data is limited [12]. We can conclude that traditional ML and DL methods have their own advantages and adaptive scenarios. Therefore, it is necessary to compare the performances of these two kinds of methods in specific task scenarios, such as in the classification of active and inactive compounds for USP7.

A study showed that effective molecular representations were crucial for ML methods, and the generalization performance of the model relied more on the choice of molecular representations than on the modeling methods [13]. The existing molecular representations are images (molecular graphs), sequences (SMILES), and values (physicochemical descriptors and molecular fingerprints) [14]. They describe compounds from different perspectives and are multimodal data with some correlations among them. Multimodal data fusion is the process of fusing different data streams [15]. Studies have shown that multimodal data fusion can help us better understand the event of interest and improve the accuracy of models [16, 17]. Currently, multimodal data fusion has been widely used in other fields, but there is a lack of exploration in ligand-based drug screening [18].

**Fig. 1:**
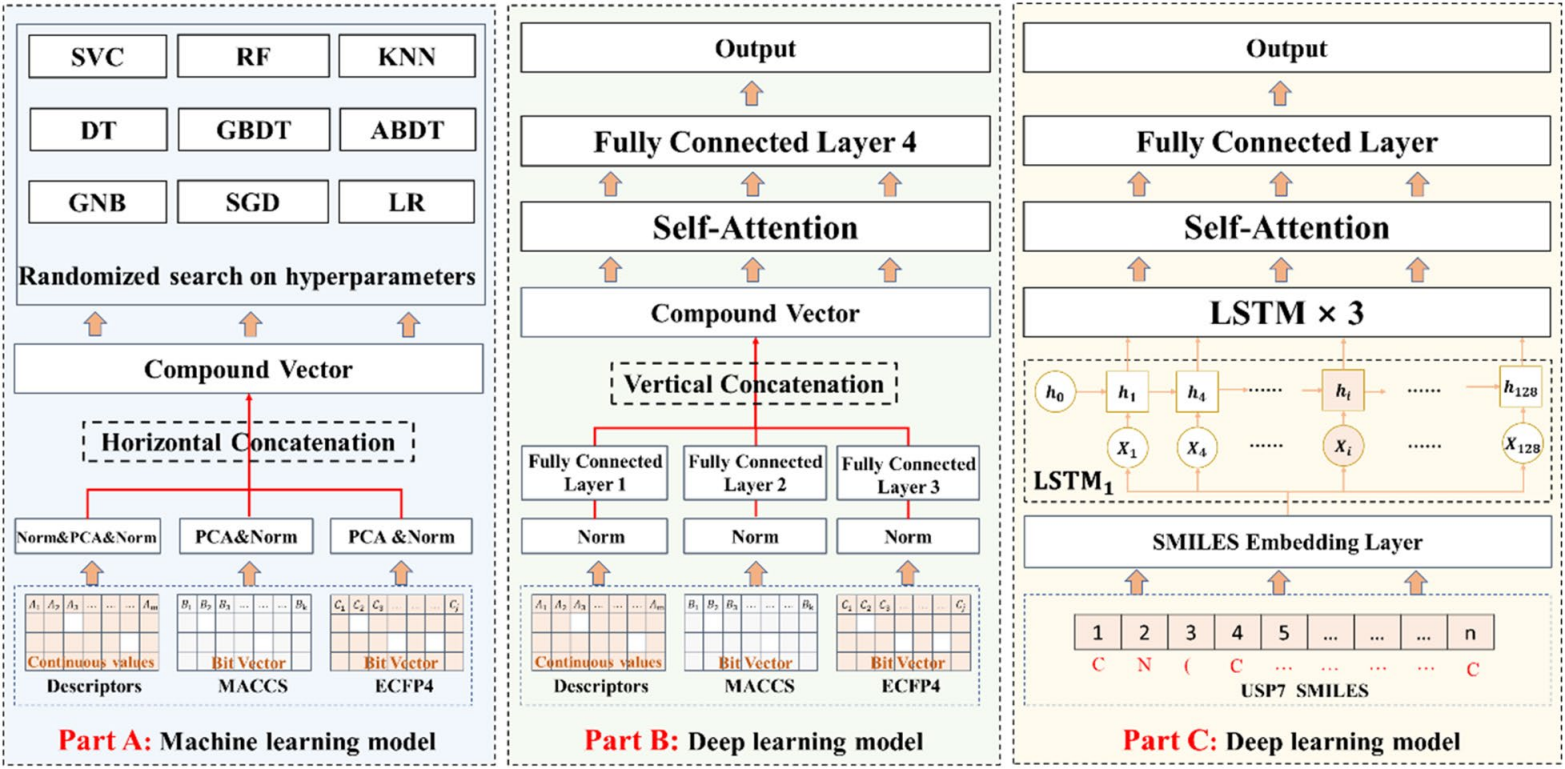
Multiple supervised binary classification models

In this study, early fusion was applied to combine physicochemical descriptors, MACCS keys and ECFP4 fingerprints to generate four new features that represented small molecules. Two deep neural networks and nine classical ML models based on four single molecular features (physicochemical descriptors, MACCS fingerprints, ECFP4 fingerprints and SMILES) and four fused features were built to distinguish active USP7 inhibitors from inactive ligands under different activity cutoff values (Fig. 1). The performance of these models was evaluated, compared and discussed using several types of metrics, which screened out the best-performing features and models in discriminating groups. SMOTE, unbiased decoy selection and SMILES enumeration were applied to improve the performance of the ML and DL models when the dataset was severely imbalanced. A dataset containing more than 4000 USP7 ligands was first constructed manually. This study has the following two objectives: (i) We established highly accurate supervised learning classification models, which would accelerate the development of USP7 inhibitors. (ii) When drug researchers apply ML and DL to ligand-based virtual screening, this study could provide some guidance for them in selecting supervised models and molecular representations as well as handling imbalanced datasets.

## Methods

### Data collection and preparation

Data of compounds with experimental K_i_, K_d_ or IC_50_ values for USP7 were collected from PubMed (https://pubmed.ncbi.nlm.nih.gov/, version: new, keywords: USP7 inhibitors), ChEMBL (https://www.ebi.ac.uk/chembl/, version: ChEMBL 29, accession ID: CHEMBL2157850) and SciFinder (https://scifinder.cas.org, version: web version, keywords: USP7 inhibitors) (accessed 13 November 2021). Specifically, the SMILES of USP7 inhibitors and related activity values were directly downloaded from ChEMBL. PubMed and SciFinder were used to search related scientific literature and patents, and then the SMILES of USP7 inhibitors and related activity values were summarized manually. Duplicates were aggregated into unique entries by the Open Babel Package (version 2.3.1, downloaded on 30 November 2021, http://openbabel.org). The experimental results with different units in the dataset were all converted into μM. The USP7 ligands in this dataset are reliable and can be traced to the source of the data. The activity cutoff value was set to 0.5 μM, 1 μM and 10 μM to distinguish the active compounds from the inactive compounds. If the activity value is less than the cutoff value, the related small molecule is an active USP7 inhibitor and regarded as positive, and its label was set to 1. Conversely, it is an inactive ligand and regarded as negative, and its label is set to 0.

#### Multiple molecular representations

The RDKit (https://www.rdkit.org/, version: 2021.03.1) was applied to convert the SMILES strings of USP7 ligands to a canonical form. A total of 208 physicochemical descriptors of compounds were calculated by RDKit. Two representative fingerprints, including the classical fingerprint MACCS keys and the best performing ECFP4 fingerprints [19, 20], were also calculated by RDKit to describe the molecular structures. These molecular representations involve three-modal molecular data, including one-dimensional sequential data (SMILES), continuous data (physicochemical descriptors) and bit vectors (fingerprints).

#### Split the dataset into training, validation and test sets

**Fig. 2:**
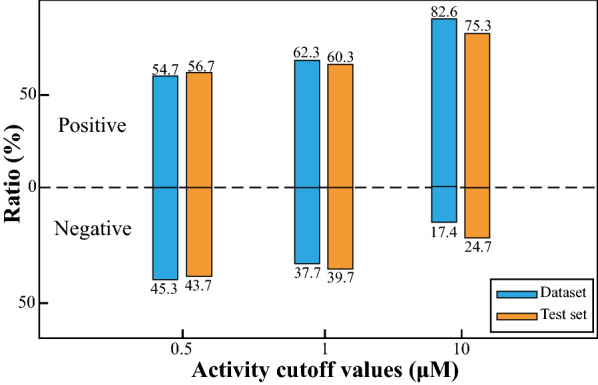
Distribution of positive and negative samples in the USP7 dataset under different activity cutoff values

To more accurately evaluate the generalization performance of the model, we clustered the dataset into 13 groups according to the small molecule structural skeleton. The clusters were obtained by applying K-means clustering analysis to the conjoint representations, which were built by concatenating the physicochemical descriptors, MACCS and ECFP4. The Calinski Harabasz Score (CHS), Davies-Bouldin index (DBI) and Silhouette Coefficient (SC) were applied as evaluation metrics to determine the most appropriate number of clusters. Detailed clustering evaluation metrics calculation procedures are described in Additional file 1, and the results are shown in Additional file 1: Fig. S1. While ensuring a similar proportion of active and inactive compounds in each dataset, 10 clusters of small molecules were selected as the training set ${\mathrm{D}}_{\mathrm{train}}$ and validation set ${\mathrm{D}}_{\mathrm{val}}$, and the remaining 3 clusters were selected as the test set ${\mathrm{D}}_{\mathrm{test}}$. As shown in Fig. 2, the test set has a proportion of positive and negative samples similar to the overall dataset. When the activity cutoff value is 0.5 μM, the overall dataset is balanced with the ratio of positive to negative samples close to 1:1. When the activity cutoff value is 1 μM and 10 μM, the overall dataset is imbalanced, with the ratio of positive and negative samples close to 3:2 and 4:1, respectively. There are 3763, 241 and 531 samples in ${\mathrm{D}}_{\mathrm{train}}$, ${\mathrm{D}}_{\mathrm{val}}$ and ${\mathrm{D}}_{\mathrm{test}}$, respectively (${\mathrm{D}}_{\mathrm{train}}$: ${\mathrm{D}}_{\mathrm{val}}$: ${\mathrm{D}}_{\mathrm{test}}$ = 83%: 5%: 12%).

### A proposal for building supervised learning models

A variety of supervised learning binary classification models, including nine ML models and two DL models, were built in this study (Fig. 1). Nine ML classifiers are comprised of support vector classification (SVC), random forest (RF), K-nearest neighbor (KNN), decision tree (DT), gradient boosting decision tree (GBDT), AdaBoost decision tree (ABDT), Gaussian naïve Bayes (GNB), stochastic gradient descent (SGD) and logistic regression (LR) (Fig. 1A). The DL classifier in Fig. 1B is constructed of a self-attention layer [21] and four fully connected layers, and the DL classifier in Fig. 1C is constructed of an embedding layer, four long short-term memory (LSTM) layers, a self-attention layer and a fully connected layer.

Physicochemical descriptors and fingerprints were preprocessed before they were used as inputs to these models. Features in physicochemical descriptors with only zero values were removed, which resulted in 194 features. Features that are measured at different scales do not contribute equally to the model fitting and model learned function and may end up creating a bias. Thus, the 194 features were then normalized by StandardScaler (*sklearn. Preprocessing,* https://scikit-learn.org), so that each feature would have mean equals 0 and the standard deviation equals 1. MACCS and ECFP4 are 166-dimensional bit vectors and 2048-dimensional bit vectors, respectively. Due to the high sparsity of the physicochemical descriptors, MACCS and ECFP4, the principal component analysis method was applied to reduce their dimensionality to 128, respectively. And detailed reasons for choosing 128 were described in the Additional file 1. Then, these three molecular representations were normalized by StandardScaler to improve the performance of the models and accelerate their convergence. All features were combined horizontally and used as input to nine ML classifiers (Fig. 1A).

For the DL model in Fig. 1B, the three molecular representations were normalized and input into fully connected layers before being combined vertically. The resulting vectors were input into a self-attention layer, and then its outputs were input into the last fully connected layer to obtain the final outputs.

In Fig. 1C, another DL model was built to process the one-dimensional sequential data SMILES. The SMILES is a chemical language and information system that represents a chemical structure by the application of a very small and natural grammar [22]. SMILES notation consists of 64 symbols, such as ‘C’, ‘N’, ‘@’, and ‘(’. As shown in Additional file 1: Fig. S2, the range of SMILES string lengths in the input is between 18 and 195 characters. SMILES with lengths less than 195 are to be padded with trailing zeros. We mapped SMILES into a vector of integer fields according to a prebuilt vocabulary, which were then input into the model. The embedding layer was applied to embed them into a continuous vector, which fully learned the relationships among the symbols in the SMILES. The embedding dimension was set to 100. A recurrent neural network (RNN) was designed for processing sequential and temporal data, such as a sentence. Long short-term memory (LSTM) networks are an extension of RNNs, which are developed to deal with the vanishing gradient problem that can be encountered when training traditional RNNs. After completing the embedding, we used the LSTM network to learn the long-term dependencies between the time steps of the sequential data. The outputs were input into a self-attention layer, and then its outputs were input into the last fully connected layer to obtain the final outputs.

### Strategies to handle imbalanced data

**Fig. 3:**
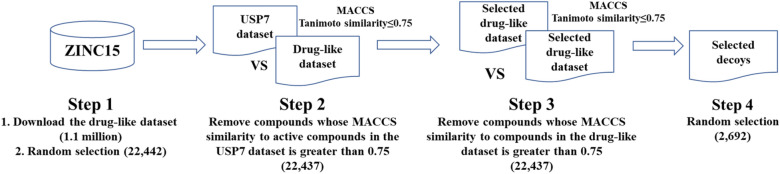
Workflow for the selection of unbiased USP7 decoy compounds

For numerical data, we applied the oversampling strategy (SMOTE) [23] and unbiased decoy selection strategy [24] to make a balanced dataset. A total of 2692 new negative samples were obtained by oversampling the negative samples in the training and validation sets. We selected unbiased decoy compounds from the ZINC database (https://zinc15.docking.org/) by referring to the literature [24]. As shown in Fig. 3, we filtered out 2692 small molecules as negative samples to balance the training and validation sets. The unbiased decoy selection strategy aims to obtain compounds that are both inactive and structurally diverse.

For sequential data, we applied the SMILES enumeration method [25] (Additional file 1: Fig. S3) and unbiased decoy selection strategy to make a balanced dataset. A total of 2692 new SMILESs were generated by enumerating the SMILESs of 656 inactive small molecules in the training and validation sets. We balanced the number of positive and negative samples only in the training and validation sets, and the test set remained unchanged.

### Experimental settings

ML algorithms use randomness when learning from a sample of data. The randomness allows the algorithm to achieve a better performing mapping of the data than if randomness is not used. Nevertheless, “randomness” requires special attention in the field of traditional ML and DL methods. The randomness may come from numerous sources, such as random splits of the dataset and random initialization of the model weights (e.g., using random seeds to initialize the model weights). To more accurately evaluate the generalization performance of the models, we used the hold-out method to split the dataset as described above. Thus, the randomness in this study came mainly from random seeds. We randomly selected 30 random seeds and calculated the mean and standard deviation (SD) of 30 experiments with these seeds to acquire objective and scientific experimental results. Specifically, we used the randomized search method [26] to find the best hyperparameter settings for each of the nine ML classifiers in 30 experiments with random seeds. The same random seeds were used in the DL models, and thus, 30 experiments with the random seeds were conducted.

#### Molecular features with different modalities

**Fig. 4:**
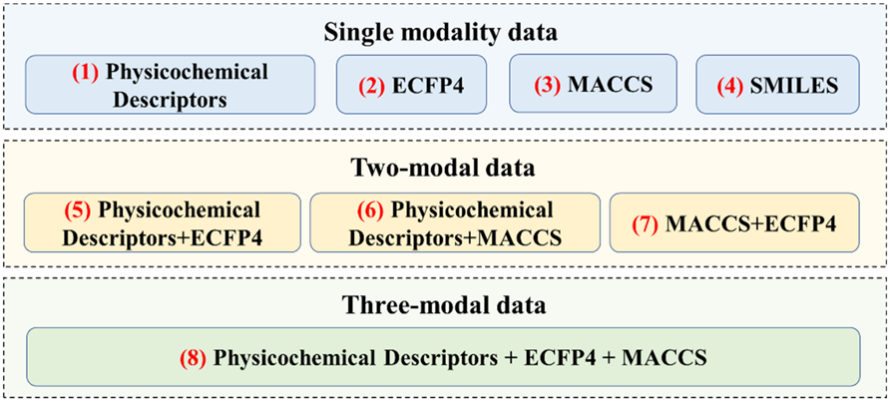
Eight molecular features with different modalities

To explore the influence of multimodal data fusion on the performance of the models, early fusion was applied to combine physicochemical descriptors, MACCS keys and ECFP4 fingerprints to generate four new features that represented small molecules (Fig. 4). A total of 15 experiments with different models based on eight features were designed, as shown in Table 1.

**Table 1:** The models and inputs of 15 experiments

Experimental index	Models	Inputs data
1	ML models	(1) Physicochemical descriptors
2		(2) ECFP4
3		(3) MACCS
4		(5) Physicochemical descriptors and ECFP4
5		(6) Physicochemical descriptors and MACCS
6		(7) MACCS and ECFP4
7		(8) Physicochemical descriptors, ECFP4 and MACCS
8	DL models	(1) Physicochemical descriptors
9		(2) ECFP4
10		(3) MACCS
11		(5) Physicochemical descriptors and ECFP4
12		(6) Physicochemical descriptors and MACCS
13		(7) MACCS and ECFP4
14		(8) Physicochemical descriptors, ECFP4 and MACCS
15	DL models	(4) SMILES

#### The settings of ML experiments

To explore the influence of molecular representations and multimodal data fusion on the performance of the supervised ML classifiers, Experiments 1–7 with nine ML models based on seven features were conducted (Table 1). Among these experiments, the input data was the only variable. The *sklearn.RandomizedSearchCV* method with 5 folds was used to find the best hyperparameters for the ML models, and the *n_iter* was set to 10. There are different hyperparameter distributions for different ML classifiers (Additional file 1: Table S3), and the specific hyperparameters for each ML model can be found at https://github.com/Hovi123123/Open-source/tree/master/results/ML_results.

To further investigate the influence of three cutoff values (0.5 μM, 1 μM and 10 μM) on the performance of nine ML models, we set up the following three sets of experiments (Table 2, Group I–Group III). As the ML models did not work very well with severely imbalanced datasets (cutoff value = 10 μM), we designed another two sets of experiments (Table 2, Groups IV and V).

**Table 2:** Five groups of experiments based on experiments 1–7 with different cutoff values and imbalanced data processing strategies

Group no.	Experiment no.	Experimental content
I	1–7	Cutoff value = 0.5 μM
II		Cutoff value = 1 μM
III		Cutoff value = 10 μM
IV		Cutoff value = 10 μM and SMOTE
V		Cutoff value = 10 μM and unbiased decoy selection

#### The settings of DL experiments

To explore the influence of multimodal data fusion on the performance of the DL models, Experiments 8–14 with a DL model based on seven features were conducted (Fig. 1B and Table 1). In addition, a DL model based on SMILES was built in Experiment 15 (Fig. 1C and Table 1). PyTorch (https://pytorch.org/) was used as the DL framework, and the detailed hyperparameter settings are shown in Additional file 1: Tables S4–S9. This study was conducted with an NVIDIA Tesla V100-DGXS-16GB.

To further investigate the influence of three cutoff values (0.5 μM, 1 μM and 10 μM) on the performance of DL models, we set up three sets of experiments (Table 3, group VI–group VIII; Table 4, group XI–group XIII). As the DL models did not work very well with severely imbalanced datasets (cutoff value = 10 μM), we designed another two sets of experiments (Table 3, groups IX and X; Table 4, groups XIV and XV).

**Table 3:** Five groups of experiments based on experiments 8–14 with different cutoff values and imbalanced data processing strategies

Group no.	Experiment no.	Experimental content
VI	8–14	Cutoff value = 0.5 μM
VII		Cutoff value = 1 μM
VIII		Cutoff value = 10 μM
IX		Cutoff value = 10 μM and SMOTE
X		Cutoff value = 10 μM and unbiased decoy selection

**Table 4:** Five groups of experiments based on experiment 15 with different cutoff values and imbalanced data processing strategies

Group no.	Experiment no.	Experimental content
XI	15	Cutoff value = 0.5 μM
XII		Cutoff value = 1 μM
XIII		Cutoff value = 10 μM
XIV		Cutoff value = 10 μM and SMILESE enumeration
XV		Cutoff value = 10 μM and unbiased decoy selection

#### Evaluation metrics

Three classification evaluation metrics, including accuracy (ACC), F1 score and area under the receiver operating characteristic curve (AUC), were applied to evaluate the model performance. Detailed information is shown in Additional file 1.

## Results and discussion

### Dataset analysis

**Fig. 5:**
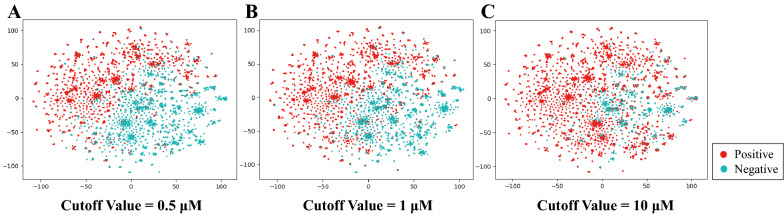
The t-SNE method was used to visualize the chemical distribution of USP7 ligands under cutoff values of 0.5 μM (**A**), 1 μM (**B**) and 10 μM (**C**) according to physicochemical descriptors and ECFP4 and MACCS keys

To investigate the possible relationships between USP7 molecular structure and classification tasks, the chemical space distribution of USP7 datasets under different activity cutoff values was analyzed. T-distributed stochastic neighbor embedding (t-SNE) was used to decompose the physicochemical descriptors, ECFP4 and MACCS keys into two dimensions for visualization. Detailed processes are described in “Using t-SNE to Visualize USP7 DataSets” in Additional file 1. As shown in Fig. 5, the positive and negative samples of USP7 datasets with an activity cutoff value of 0.5 μM occupy specific regions that are well separated. However, as the cutoff value increases, the degree of dataset imbalance increases, and the distribution boundaries of positive and negative samples become weaker. Thus, the ML models will theoretically become more difficult to differentiate between positive and negative samples.

### Performance evaluation of nine ML models

This section aims to investigate the influence of different modalities as well as activity cutoff values on the performance of nine ML models. Meanwhile, two strategies, including SMOTE and unbiased decoy selection, were applied to deal with severely imbalanced data. A *t*-test was used to determine if there was a significant difference between the means of the evaluation metrics for the two models, and the results are shown in Additional file 1: Table S10.

#### Performance evaluation of ML on different single modality data

##### Balanced dataset

**Fig. 6:**
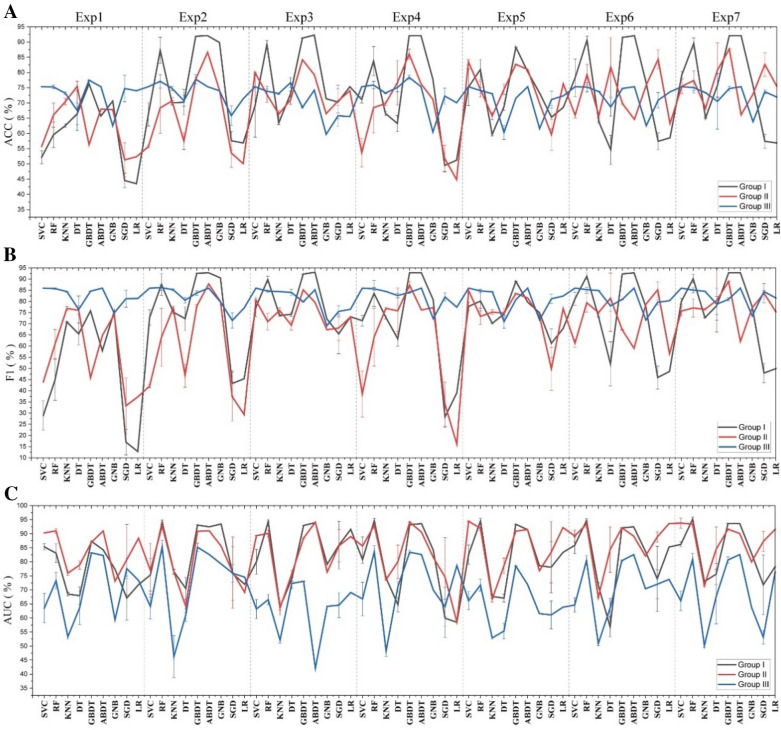
The ACC (**A**), F1 (**B**) and AUC (**C**) of nine ML models on the test set in Groups I, II and III (mean ± standard deviation (SD), %). *Exp means the experiment no. in each group

When the activity cutoff value is set to 0.5 μM, the overall dataset is balanced with the ratio of positive to negative samples close to 1:1. As shown in Fig. 6 (Group I, exp1–exp3), nine supervised ML algorithms were applied to construct classifiers for physicochemical descriptors, ECFP4 or MACCS, yielding a total of 27 classifiers for distinguishing USP7 active and inactive small molecules. The performance of these models varied widely on the test set, with ACC ranging from 43.50 to 92.28%, F1 ranging from 12.79 to 93.02%, and AUC ranging from 63.93 to 94.31% (Additional file 1: Table S11). It was not hard to find that MACCS-based ABDT performed the best.

Different molecular representations depict compounds from diverse perspectives, which are suitable for different ML algorithms. Different ML algorithms also favor different data structures. As shown in Additional file 1: Tables S10 and S11, in terms of molecular representations, the evaluation metrics of ML models could be generally ranked from best to worst as follows: MACCS > ECFP4 > Physicochemical descriptors, except that GNB, KNN and GBDT based on ECFP4 performed significantly better than on MACCS and on physicochemical descriptors. It was not surprising that the ensemble classifiers (ABDT, GBDT and RF) generally outperformed the other single classifiers (Fig. 6). Among all the single classifiers, ECFP4-based GNB performed the best and was comparable to the MACCS-based and ECFP4-based ensemble models (Fig. 6).

##### Slightly imbalanced dataset

When the activity cutoff value is 1 μM, the overall dataset is slightly imbalanced, with a ratio of positive to negative samples close to 3:2. As shown in Fig. 6 (Group II, exp1–exp3), nine supervised ML algorithms were applied to construct classifiers for physicochemical descriptors, ECFP4 or MACCS, when the activity value threshold was 1 μM, yielding a total of 27 classifiers for distinguishing USP7 active and inactive small molecules. As shown in Additional file 1: Table S10 and S11, in terms of molecular representations, the performance ranking of ML models trained on the slightly imbalanced dataset is almost identical to the ML models trained on the balanced dataset: MACCS > ECFP4 > physicochemical descriptors, except that SVC, DT and LR based on physicochemical descriptors perform significantly better than those based on ECFP4, and KNN, ABDT and GNB based on ECFP4 perform significantly better than those based on MACCS. Among the 27 models, the ECFP4-based ABDT performs the best, with its evaluation metrics all approximately 90.00%, only slightly inferior to the best-performing model trained on the balanced dataset.

As shown in Fig. 6, Additional file 1: Tables S10 and S11, the performance of ML classifiers trained on slightly imbalanced datasets is mostly (16/27) significantly poorer (with at least two evaluation metrics, the same below) than the performance of ML classifiers trained on balanced datasets. However, most of the physicochemical descriptor-based ML models trained on slightly imbalanced datasets perform better, except for GBDT and GNB. Nevertheless, DT, as the best-performing physicochemical descriptors-based ML model trained on slightly imbalanced datasets, shows significantly poorer performance than physicochemical descriptor-based GBDT trained on balanced datasets. Almost all the ECFP4-based ML models perform significantly better on the balanced dataset, except for the KNN model, which performs significantly better on the slightly imbalanced dataset than on the balanced dataset. Most of the MACCS-based ML models (6/9) perform significantly better on balanced datasets, but SVC and KNN perform significantly better on slightly imbalanced datasets, and SGD performs similarly in both cases. The ensemble classifiers ABDT and GBDT on the slightly imbalanced dataset still outperform the other single classifiers. But the performance of ensemble classifier RF is medium. The MACCS-based SVC performs the best among all the single classifiers, but its performance is still significantly poorer than the optimal ensemble classifier.

##### Severely imbalanced dataset

When the activity cutoff value is 10 μM, the overall dataset is severely imbalanced, with a ratio of positive to negative samples close to 4:1. Since AUC is a better metric for imbalanced classification compared to other metrics, we evaluate the performance of the model with AUC as the main metric and other metrics as supplementary information. As shown in Fig. 6 (Group III, exp1–exp3), nine supervised ML algorithms were applied to construct classifiers for physicochemical descriptors, ECFP4 or MACCS, when the activity value threshold was 10 μM, yielding a total of 27 classifiers for distinguishing USP7 active and inactive small molecules. The performance of these models varied widely on the test set, with ACC ranging from 59.70 to 77.80%, F1 ranging from 69.08 to 86.15%, and AUC ranging from 42.11 to 85.36% (Additional file 1: Table S12). The performance of ML classifiers decreases with increasing dataset imbalance. Among the 27 models, 25 ML classifiers based on severely imbalanced datasets showed lower performance than the classifiers based on balanced datasets, and 26 ML classifiers based on severely imbalanced datasets indicated lower performance than the classifiers based on slightly imbalanced datasets. However, the ECFP4-based LR performed significantly better on the severely imbalanced dataset than on both the slightly imbalanced and balanced datasets. This finding suggests that LR based on ECFP4 is more suitable for handling imbalanced datasets compared to balanced datasets.

In terms of molecular representations, the performance ranking of ML models trained on the severely imbalanced dataset is different from the performance ranking of ML models trained on the slightly imbalanced and balanced dataset: ECFP4 > physicochemical descriptors > MACCS, except that SGD and LR based on physicochemical descriptors perform significantly better than the models based on ECFP4, and DT based on MACCS performs the best among the three DT models (Fig. 6, Additional file 1: Tables S10 and S12). The ensemble classifiers ABDT, GBDT and RF on the severely imbalanced dataset generally outperformed the other single classifiers, indicating that the ensemble models are suitable for handling imbalanced datasets. However, the performance of these ensemble models on the severely imbalanced dataset decreases significantly compared to the balanced and slightly balanced datasets (Fig. 6, Additional file 1: Tables S10 and S12). Among all the single classifiers, the ECFP4-based GNB performs the best with all the evaluation metrics below 80.00%, but its performance is still significantly poorer than the optimal ensemble classifier. It was not hard to find that the performance of the ECFP4-based RF was the best, with an AUC and F1 above 85.00% but an ACC of only 77.11%, which is significantly worse than the best model trained on the balanced and slightly balanced datasets.

**Fig. 7:**
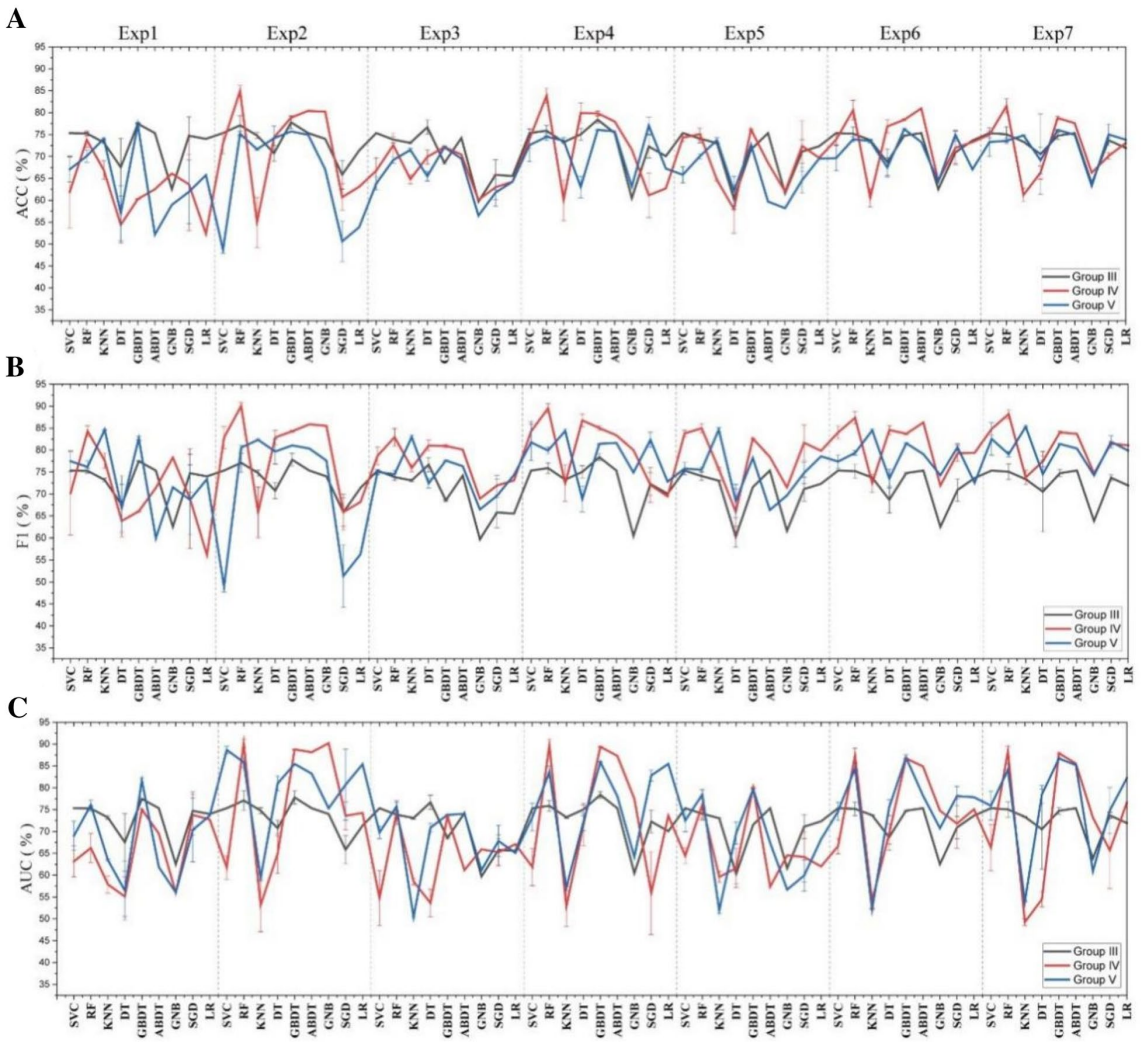
The ACC (**A**), F1 (**B**) and AUC (**C**) of nine ML models on the test set in Groups III, IV and V (mean ± SD, %). *Exp means the experiment no. in each group

To improve the performance of the ML models trained on the severely imbalanced dataset, we applied the SMOTE and unbiased decoys selection strategy to make a balanced dataset. Then, the performance of the ML models based on the new dataset was evaluated on the original severely imbalanced test set, and the results are shown in Fig. 7 (Groups IV and V). Both strategies can improve the AUC values of some models to some extent. Among the 27 models, the SMOTE strategy was able to improve the AUC values of 13 ML models, and the unbiased decoy selection strategy could improve the AUC values of 17 ML models. The ECFP4-based RF with the SMOTE strategy has the best performance, with an AUC and F1 over 90.00% and an ACC of 84.94%, which is close to the best model trained on the balanced dataset. Therefore, we recommend these two strategies to improve the performance of ML models when we encounter an imbalanced dataset.

#### Performance evaluation of ML on multimodal data

##### Balanced dataset

To improve the performance of the classification models trained on balanced datasets, a multimodal data fusion strategy is tentatively employed to construct ML models. Four new fused features based on physicochemical descriptors, ECFP4 and MACCS, were acquired, as shown in Fig. 4, and then they were applied as inputs to the nine ML models. The performance of the 36 ML models is shown in Fig. 6 and Additional file 1: Table S11 (Group I, Exp 4–7). Among these models, eight models based on fused features performed better than the models based on individual features. The best-performing model based on fused features is GBDT based on feature (8), whose evaluation metrics are all above 92.00%. However, its performance is still significantly poorer than MACCS-based ABDT, which indicates that multimodal data fusion cannot improve the model performance. We speculated that the performance of ML models based on a single molecular representation was too good to improve when the dataset was balanced.

##### Slightly imbalanced dataset

To improve the performance of the classification models trained on slightly imbalanced datasets, a multimodal data fusion strategy is tentatively employed to construct ML models. Among the 36 ML models in Fig. 6 and Additional file 1: Table S11 (Group II, Exp 4–7), 12 models based on fused features performed better than the models based on individual features. The best-performing model based on fused features is GBDT based on feature (8), whose evaluation metrics are all above 87.00%. Its performance is significantly better than the performance of the ECFP4-based ABDT, which indicates that multimodal data fusion can improve the model performance when the dataset is slightly imbalanced.

##### Severely imbalanced dataset

To improve the performance of the classification models trained on severely imbalanced datasets, a multimodal data fusion strategy is tentatively employed to construct ML models. Among the 36 ML models in Fig. 6 and Additional file 1: Table S12 (Group III, Exp 4–7), 5 models based on fused features performed better than the models based on individual features. The best-performing model based on fused features is RF based on feature (5), whose performance is significantly poorer than the best-performing ECFP4-based RF trained on the severely imbalanced dataset, which indicates that multimodal data fusion cannot improve the model performance.

To improve the performance of the multimodal data-based ML models trained on the severely imbalanced dataset, we applied the SMOTE and unbiased decoy selection strategy to make a balanced dataset. Then, the performance of the ML models based on the new dataset was evaluated on the original severely imbalanced test set, and the results are shown in Fig. 7 and Additional file 1: Table S12 (Groups IV and V, exp4–exp7). Compared to the models with multimodal data as input (Group III, exp4–exp7, a total of 36 models), the SMOTE strategy was able to improve the AUC values of 8 ML models (Group IV, exp4–exp7, a total of 36 models), and the unbiased decoy selection strategy could significantly improve the AUC values of 6 ML models (Group V, exp4–exp7, a total of 36 models). The feature (5)-based RF with the SMOTE strategy has the best performance, whose performance is significantly poorer than the best-performing ECFP4-based RF with the SMOTE strategy, which indicates that multimodal data fusion cannot further improve the model performance.

#### Measuring the stability of ML algorithms

The stability of the model is also a very important point, which is affected mainly by the random seeds used in the models here. The optimal hyperparameters found by the randomized search method with different random seeds vary widely, so the model results fluctuate somewhat. As shown in Figs. 6, 7, Additional file 1: Tables S11 and S12, SVC, RF, KNN, DT, GBDT and SGD showed lower stability, with SDs ranging from 0.00 to 14.85%, while ABDT, GNB and LR were stable classifiers, with SDs equal to 0.00. Random seeds affect the initialization of SVC, DT and SGD or splitting operations, etc. The optimal hyperparameters with different random seeds thus vary, which makes SVC, DT and SGD unstable. KNN does not have the parameter of random seeds, and the fluctuation of the KNN algorithm is due to the change in hyperparameters caused by the randomized search method. The performance of GNB is very stable because random seeds are not present in its parameters. Meanwhile, since we set the parameter “solver” of LR to the default option “lbfgs”, this will make it not shuffle the data, i.e., it will not be affected by random seeds.

Although ensemble classifiers performed well, RF was not very stable, with SD ranging from 0.34 to 13.02%. GBDT showed relatively stable performance, with SD ranging from 0.00 to 1.68%, and ABDT was very stable, with SD equal to 0.00. This phenomenon can be explained by the following aspects: (1) RF uses feature bagging to create an ensemble learner in which each DT is used as a parallel estimator. Each DT takes a subsample from the entire dataset and gives a class prediction. The class with the most votes becomes the prediction of our model. The different data divisions caused by random seeds could result in different prediction results of DT, which thus affects the stability of the final results. (2) GBDT and ABDT are both built using a method called boosting in which each DT is connected sequentially to obtain a strong learner. Because there is no need for creating subsamples from the dataset, their optimal hyperparameters do not change at all under different random seeds, which results in stable performance of models.

### Performance evaluation of DL methods

#### Performance evaluation of DL on different modal data

##### Balanced dataset

**Fig. 8:**
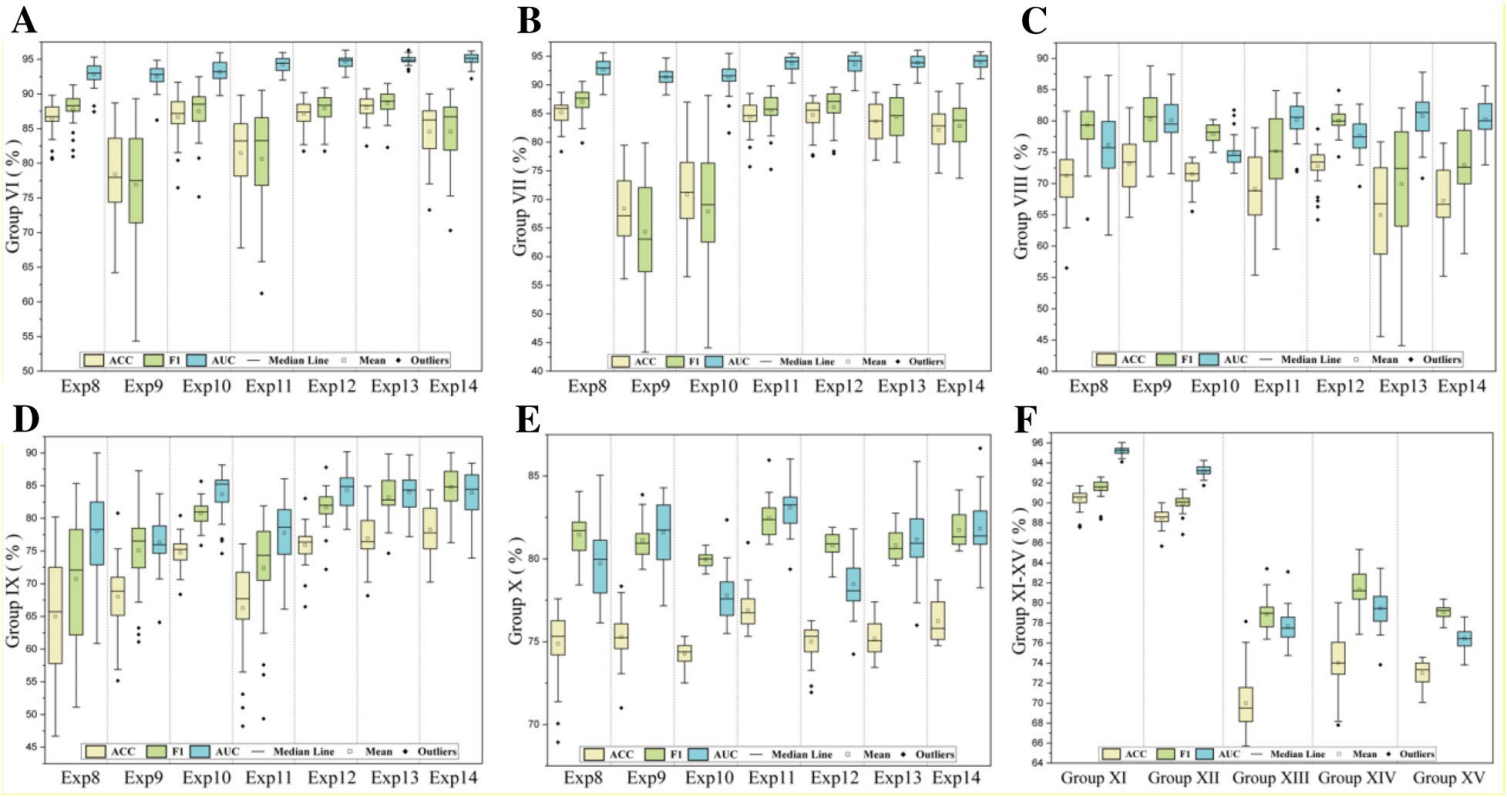
The evaluation metrics of DL models with group no. of VI (**A**), VII (**B**), VIII (**C**), IX (**D**), X (**E**) and XI–XV (**F**) on the test set (mean ± SD, %). *Exp means the experiment no. in each group

When the activity cutoff value is set to 0.5 μM, the overall dataset is balanced with the ratio of positive to negative samples close to 1:1. As shown in Fig. 8A (GroupVI, exp8–exp14), the DL framework in Fig. 1B was applied to construct classifiers for the seven features in Fig. 4, yielding a total of 7 classifiers for distinguishing USP7 active and inactive small molecules. The performance of these models varied widely on the test set, with ACC ranging from 78.40 to 88.05%, F1 ranging from 76.91 to 88.68%, and AUC ranging from 92.46 to 94.92% (Additional file 1: Table S13). In terms of molecular representations, the performance of the DL models could be generally ranked from best to worst as follows: MACCS + ECFP4 > Physicochemical descriptors + MACCS > MACCS > Physicochemical descriptors > Physicochemical descriptors + MACCS + ECFP4 > Physicochemical descriptors + ECFP4 > ECFP4. This result indicated that multimodal data fusion can improve the performance of the DL model. The DL model based on MACCS + ECFP4 has the best performance, with evaluation metrics all above 88% and an AUC of 94.87%.

##### Slightly imbalanced dataset

When the activity cutoff value is 1 μM, the overall dataset is slightly imbalanced, with a ratio of positive to negative samples close to 3:2. As shown in Fig. 8B (Group VII, exp8–exp14), the DL framework in Fig. 1B was applied to construct classifiers for the seven features in Fig. 4 when the activity value threshold was 1 μM, yielding a total of 7 classifiers for distinguishing USP7 active and inactive small molecules. The performance of these models varied widely on the test set, with ACC ranging from 68.39 to 85.22%, F1 ranging from 64.33 to 87.07%, and AUC ranging from 91.37 to 93.92% (Additional file 1: Table S13). Since AUC is a better metric for imbalanced classification compared to other metrics, we evaluate the performance of the model with AUC as the main metric and other metrics as supplementary information. In terms of molecular representations, the performance of the DL models trained on the slightly imbalanced dataset could be ranked from best to worst as follows: physicochemical descriptors + MACCS + ECFP4 > MACCS + ECFP4 > physicochemical descriptors + ECFP4 > physicochemical descriptors + MACCS > physicochemical descriptors > MACCS > ECFP4. This result indicated that multimodal data fusion could improve the performance of the DL model. The DL model based on physicochemical descriptors + MACCS + ECFP4 has the best performance, with evaluation metrics all above 82.00% and an AUC of 93.92%. However, the performance of this model is still significantly inferior to the optimal DL model based on a balanced dataset.

##### Severely imbalanced dataset

When the activity cutoff value is 10 μM, the overall dataset is severely imbalanced, with a ratio of positive to negative samples close to 4:1. As shown in Fig. 8C (Group VIII, exp8–exp14), the DL framework in Fig. 1B was applied to construct classifiers for the seven features in Fig. 4 when the activity value threshold was 10 μM, yielding a total of 7 classifiers for distinguishing USP7 active and inactive small molecules. The performance of these models varied widely on the test set, with ACC ranging from 64.98 to 73.11%, F1 ranging from 69.91 to 80.24%, and AUC ranging from 74.98 to 80.78% (Additional file 1: Table S14). In terms of molecular representations, the performance of the DL models trained on the severely imbalanced dataset could be ranked from best to worst as follows: MACCS + ECFP4 > physicochemical descriptors + MACCS + ECFP4 > physicochemical descriptors + ECFP4 > ECFP4 > physicochemical descriptors + MACCS > physicochemical descriptors > MACCS. This result indicated that multimodal data fusion can improve the performance of the DL model. The DL model based on MACCS + ECFP4 has the best performance, with an AUC of 80.78% but ACC and F1 below 70.00%. The performance of this model is significantly worse than the performance of the optimal DL model based on a balanced or slightly imbalanced dataset.

To improve the performance of the DL models trained on the severely imbalanced dataset, we applied the SMOTE and unbiased decoys selection strategy to make a balanced dataset. Then, the performance of the DL models based on the new dataset was evaluated on the original severely imbalanced test set, and the results are shown in Fig. 8D, E (Group IX and X). Both strategies can improve the AUC values of some models to some extent. Among the 7 models, the SMOTE strategy was able to improve the AUC values of 5 DL models, and the unbiased decoy selection strategy could improve the AUC values of 7 DL models. The SMOTE strategy can increase the AUC of the model in Group VIII up to 84.32%, while the unbiased decoy selection strategy can only increase the AUC of the model in Group VIII up to 83.09%. Both methods were able to improve ACC and F1 to more than 75.00%. The ECFP4-based DL with SMOTE strategy has the best performance, with evaluation metrics of approximately 80.00%. Although the performance of this model is still significantly poorer than the best DL model trained on a balanced dataset, the improvement of its performance is significantly obvious compared with the best DL model in Group VIII. Therefore, we recommend these two strategies to improve the performance of the DL models when we encounter an imbalanced dataset.

#### Performance evaluation of DL on SMILES

We designed three groups of experiments (Group XI–XIII) based on Exp 15 to explore the performance of the SMILES-based DL model trained on the balanced dataset, slightly imbalanced dataset and severely imbalanced dataset. The results are shown in Fig. 8F and Additional file 1: Table S15. The DL model trained on the balanced dataset has the best performance (Group XI), with evaluation metrics all over 90.00% and an AUC of 95.20%. As the degree of dataset imbalance gradually increases, the performance of DL classifiers decreases. When the dataset is slightly imbalanced, the performance of the DL model is still good, with ACC and F1 close to 90.00% and AUC of 93.21% (GroupXII). When the dataset is severely imbalanced, the performance of the DL model is not that good, with evaluation metrics below 80.00% (Group XIII).

To improve the performance of the DL models trained on the severely imbalanced dataset, we applied the SMILES enumeration and unbiased decoys selection strategy to make a balanced dataset. Then, the performance of the DL models based on the new dataset was evaluated on the original severely imbalanced test set, and the results are shown in Fig. 8F (Groups XIV and XV). Both strategies can improve the AUC values of some models to some extent. The SMILES enumeration strategy performed significantly better than the unbiased decoy selection strategy. Specifically, the SMILES enumeration strategy improves the AUC value of the DL model in Group XIII from 77.70 to 79.47%, while the unbiased decoy selection strategy does not improve the AUC value of the DL model. Therefore, we recommend the SMILES enumeration strategy to improve DL model performance when dealing with imbalanced datasets. Although the performance of the DL model was significantly improved through the above two strategies, its performance was still significantly poorer than the performance of the best DL model trained on a balanced dataset.

### Comparison of DL and ML models

The optimal ML and DL models under different cutoff values are shown in Table 5 below. The optimal models and the remaining models under the same activity cutoff value perform significantly different (Additional file 1: Table S10). According to the data in Table 5 and Additional file 1: Table S10, classifiers with significant advantage will be recommended for different activity cutoff values. When the dataset is balanced, we recommend the MACCS-based ABDT rather than the optimal DL models. When the dataset is slightly imbalanced, we recommend the SMILES-based DL model. When the dataset is severely imbalanced, we recommend using the SMOTE strategy to balance the dataset and construct the RF model based on ECFP4. The distribution boundaries between positive and negative samples almost disappear when the threshold value is 10 μM (Fig. 5), which makes the model theoretically difficult to differentiate between positive and negative samples. Therefore, it is reasonable that the ML and DL models trained on severely imbalanced datasets show poorer performance than those trained on balanced datasets.

**Table 5:** Performance evaluation metrics of the optimal ML model and DL model with different activity cutoff values

Cutoff value	Model	ACC	F1	AUC
	ABDT based on MACCS	92.28 ± 0.00	93.02 ± 0.00	93.94 ± 0.00
0.5 μM	DL based on MACCS + ECFP4 (rank 1)	88.05 ± 1.78	88.68 ± 1.98	94.87 ± 0.62
	DL based on physicochemical descriptors + MACCS (rank 2)	87.14 ± 1.93	87.93 ± 2.15	94.69 ± 0.89
	DL based on SMILES	90.40 ± 0.94	91.47 ± 0.93	95.20 ± 0.43
	GBDT based on physicochemical descriptors + ECFP4 + MACCS	87.68 ± 0.09	88.88 ± 0.10	91.59 ± 0.16
1 μM	DL based on physicochemical descriptors + ECFP4 + MACCS (rank 1)	82.13 ± 3.54	82.83 ± 4.08	93.92 ± 1.40
	DL based on MACCS + ECFP4 (rank 2)	83.62 ± 3.54	84.44 ± 3.95	93.89 ± 1.43
	DL based on SMILES	88.48 ± 0.88	89.92 ± 0.94	93.21 ± 0.54
	RF based on ECFP4 and SMOTE	84.94 ± 1.25	90.04 ± 0.84	90.20 ± 0.97
10 μM	DL based on physicochemical descriptors + MACCS and SMOTE (rank 1)	75.91 ± 2.99	81.68 ± 2.73	84.32 ± 3.17
	DL based on ECFP4 + MACCS and SMOTE (rank 2)	76.98 ± 3.84	83.23 ± 3.35	84.00 ± 2.96
	DL based on MACCS and SMOTE (rank 4)	74.80 ± 2.40	80.80 ± 1.96	83.70 ± 3.43
	DL based on physicochemical descriptors + ECFP4 and unbiased decoy selection (rank 5)	76.90 ± 1.29	82.45 ± 1.18	83.09 ± 1.37
	DL based on SMILES and SMILES enumeration	74.02 ± 3.08	81.34 ± 1.93	79.47 ± 2.07

Although the model selection is more a case-by-case analysis, there are still some rules learned from this study. While ML models are easier to construct using the *sklearn* package, DL methods must be constructed manually from scratch and continuously adjust the network structure and hyperparameter settings. In addition, when dealing with balanced datasets, ML models tend to be more accurate and efficient than DL models. When dealing with imbalanced datasets, SMOTE, unbiased decoy selection, SMILES enumeration and multimodal data infusion strategies may improve the performance of both ML and DL models.

**Fig. 9:**
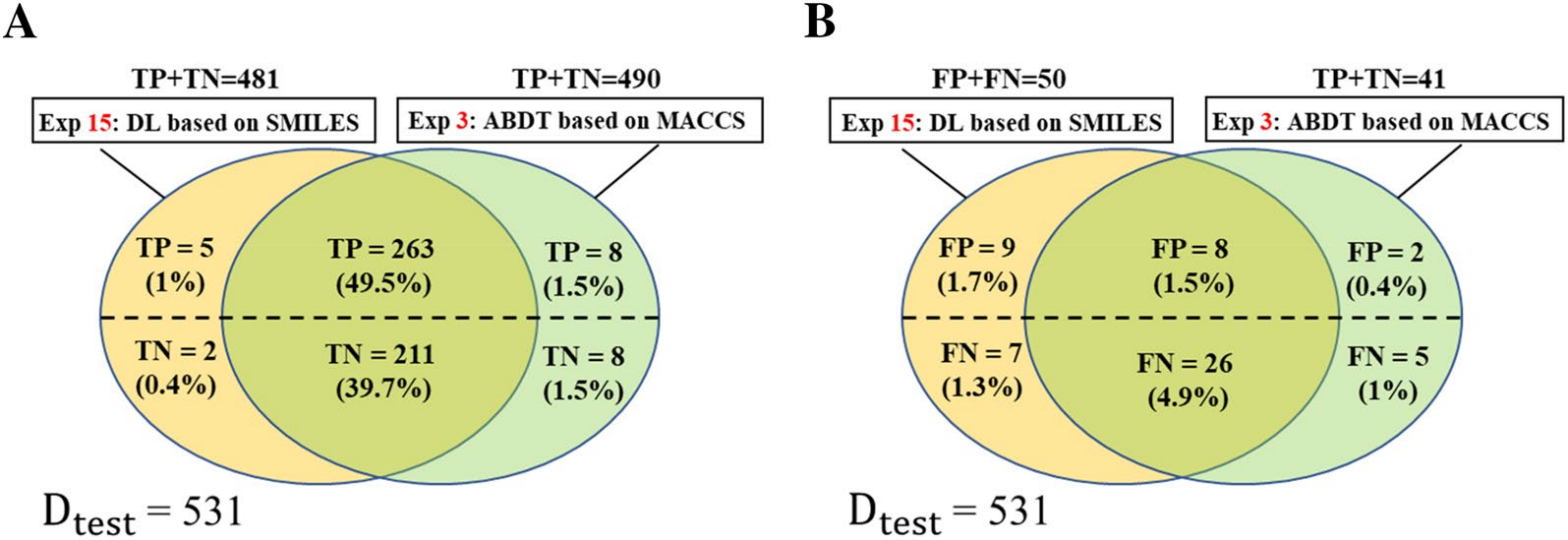
Venn diagram showing the number and percentage of correct predictions (**A**) and incorrect predictions (**B**) shared between DL based on SMILES and ABDT based on MACCS

When the dataset is balanced, the MACCS-based ABDT model significantly performs the best among all ML models, and the SMILES-based DL model significantly outperforms all the other DL models (Table 5 and Additional file 1: Table S10). We then compared the TP, FP, TN and FN identified by the MACCS-based ABDT and SMILES-based DL models on the same test set with 531 samples, which showed that the prediction results of the two models mostly overlapped (95.6%) in Fig. 9. To provide more about the classifier differences highlighted by Venn diagrams in Fig. 9, we depicted related molecular structures in Additional file 1: Figs. S4 and S5, which showed that different skeletal structures fell to different parts of the Venn diagram.

## Conclusions

In this study, early fusion was applied to combine physicochemical descriptors, MACCS keys and ECFP4 fingerprints to generate four new features that represented small molecules. Two DL models and nine classical ML models based on four single molecular features (physicochemical descriptors, MACCS keys, ECFP4 fingerprints and SMILES) and four fused features were built to distinguish active USP7 inhibitors from inactive ligands under three activity cutoff values. The optimal models are ensemble learning models when the dataset is balanced or severely imbalanced, and SMILES-based DL performs the best when the dataset is slightly imbalanced. Multimodal data fusion in some cases can improve the performance of ML and DL models. In addition, SMOTE, unbiased decoy selection and SMILES enumeration can improve the performance of ML and DL models when the dataset is severely imbalanced, and SMOTE works the best. We established an in-house dataset of USP7 small molecule inhibitors. To the best of our knowledge, this is the largest dataset of USP7 inhibitors, which may help researchers carry out USP7 inhibitor-related work. Our study established highly accurate supervised learning classification models, which would provide alternative options for screening USP7 inhibitors in addition to conventional structure-based and ligand-based methods.

## Supplementary Information

**Additional file 1: Figure S1.** Statistics of data clustering results. **Figure S2.** Histogram showing the length of SMILES string in the USP7 targeted small molecules. **Table S1.** The evaluation metrics of the RF classifiers on test set. **Table S2.** The evaluation metrics of the DL model on test set. **Table S3.** Hyperparameters of ML models considered in optimization. **Tables S4–S8.** Hyperparameter settings of experiments 8–14 in Group VI-X. **Table S9.** Hyperparameter settings of experiment 15 in Group XI-XV. **Figure S3.** SMILES enumeration of aspirin, the left is the canonical SMILES. **Table S10.** t-test results. **Table S11.** The evaluation metrics of the ML models on test set in Group I and II (mean ± SD, %). **Table S12.** The evaluation metrics of the ML models on test set in Group III-V (mean ± SD, %). **Table S13.** The evaluation metrics of the DL models on test set in Group VI and VII (mean ± SD, %). **Table S14.** The evaluation metrics of the DL models on test set in Group VIII-X (mean ± SD, %). **Table S15.** The evaluation metrics of the DL models on test set in Group XI-XV (mean ± SD, %). **Figure S4.** The classifier differences highlighted by Venn diagrams in Fig. 9A. **Figure S5.** The classifier differences highlighted by Venn diagrams in Fig. 9B.

## Data Availability

The source codes and dataset are freely available for use and download at the Github repository: https://github.com/Hovi123123/Open-source. The Git tag is v1.0.0.

## Abbreviations

USP7: Ubiquitin-specific-processing protease 7
ML: Machine learning
DL: Deep learning
SMILES: Simplified molecular input line entry system
SVC: Support vector classification
RF: Random forest
KNN: K-NearestNeighbor
DT: DecisionTree
GBDT: Gradient boosting decision tree
ABDT: AdaBoost decision tree
GNB: Gaussian Naive Bayes
SGD: Stochastic gradient descent
LR: Logistic regression
RNN: Recurrent neural network
LSTM: Long short-term memory
ACC: Accuracy
AUC: Area under receiver operating characteristic curve
SMOTE: Synthetic minority over-sampling technique
