# Supplementary material for: Multimodal data fusion for supervised learning-based identification of USP7 inhibitors: a systematic comparison
Source: J Cheminform. 2023 Jan 11;15:5. doi: 10.1186/s13321-022-00675-8 (PMC9835315; doi:10.1186/s13321-022-00675-8)
Supplement: Supplementary file 1 — Additional file 1: Figure S1. Statistics of data clustering results. Figure S2. Histogram showing the length of SMILES string in the USP7 targeted small molecules. Table S1. The evaluation metrics of the RF classifiers on test set. Table S2. The evaluation metrics of the DL model on test set. Table S3. Hyperparameters of ML models considered in optimization. Tables S4–S8. Hyperparameter settings of experiments 8–14 in Group VI-X. Table S9. Hyperparameter settings of experiment 15 in Group XI-XV. Figure S3. SMILES enumeration of aspirin, the left is the canonical SMILES. Table S10. t-test results. Table S11. The evaluation metrics of the ML models on test set in Group I and II (mean ± SD, %). Table S12. The evaluation metrics of the ML models on test set in Group III-V (mean ± SD, %). Table S13. The evaluation metrics of the DL models on test set in Group VI and VII (mean ± SD, %). Table S14. The evaluation metrics of the DL models on test set in Group VIII-X (mean ± SD, %). Table S15. The evaluation metrics of the DL models on test set in Group XI-XV (mean ± SD, %). Figure S4. The classifier differences highlighted by Venn diagrams in Fig. 9A. Figure S5. The classifier differences highlighted by Venn diagrams in Fig. 9B. [file 13321_2022_675_MOESM1_ESM.docx]

Multimodal data fusion for supervised learning-based identification of USP7 inhibitors: a systematic comparison

**Wen-feng Shen^1†^, He-wei Tang ^1†^, Jia-bo Li ^1^, Xiang Li^2*^, Si Chen^1*^**

^1^School of Medicine & School of Computer Engineering and Science, Shanghai University, Shanghai 200444, China

^2^School of Pharmacy, Second Military Medical University, Shanghai 200433, China

^†^These authors have contributed equally to this work

*** Correspondence:**

Xiang Li

xiangli@smmu.edu.cn

Si Chen

caroline-sisi-chen@hotmail.com

**Table of content**

**Fig. S1** Statistics of data clustering results.

**Fig. S2** Histogram showing the length of SMILES string in the USP7 targeted small molecules. The x-axis shows the length of SMILES string and the y-axis shows the corresponding number of USP7 targeted small molecules.

The reason why the dimensionality of the physicochemical descriptors, MACCS and ECFP4 was reduced to 128 and not any other number.

**Table S1** The evaluation metrics of the RF classifiers on test set.

**Table S2** The evaluation metrics of the DL model on test set.

**Table S3** Hyperparameters of ML models considered in optimization.

**Table S4-8** Hyperparameter settings of experiments 8-14 in Group VI-X.

**Table S9** Hyperparameter settings of experiment 15 in Group XI-XV.

**Fig. S3** SMILES enumeration of aspirin, the left is the canonical SMILES.

Evaluation metrics

Using t-SNE to Visualize USP7 DataSets.

**Table S10** t-test results.

**Table S11** The evaluation metrics of the ML models on test set in Group I and II (mean ± SD, %). * Exp means the index of experiments in each groups.

**Table S12** The evaluation metrics of the ML models on test set in Group III-V (mean ± SD, %). * Exp means the index of experiments in each groups.

**Table S13** The evaluation metrics of the DL models on test set in Group VI and VII (mean ± SD, %). * Exp means the index of experiments in each groups.

**Table S14** The evaluation metrics of the DL models on test set in Group VIII-X (mean ± SD, %). * Exp means the index of experiments in each groups.

**Table S15** The evaluation metrics of the DL models on test set in Group XI-XV (mean ± SD, %). * Exp means the index of experiments in each groups.

**Fig. S4** The classifier differences highlighted by Venn diagrams in Fig. 9A.

**Fig. S5** The classifier differences highlighted by Venn diagrams in Fig. 9B.

**Clustering Evaluation Metrics**

Determining the number of clusters in a data set is an essential yet difficult step in cluster analysis. This task involves more than one criterion. Here, the Calinski Harabasz Score (CHS), Davies-Bouldin index (DBI), and Silhouette Coefficient (SC) were applied as evaluation indexes to determine the most appropriate number of clusters. CHS can reflect the compactness among clusters. The higher CHS indicates the better clustering effect. CHS is calculated by the inter-cluster and intra-cluster variance as follows:

$\mathrm{CHS}\left( k \right)=\frac{tr(B_{k})}{tr(W_{k})}\frac{N-k}{k-1}$ (1)

$k$ is the number of clusters we manually select and $N$ is the number of objects in the data set.$B_{k}$ and $W_{k}$ denote the inter-cluster and intra-cluster sums of squares respectively , which are calculated as follows:

$B_{k}=\sum_{q} n_{q}(c_{q}-c){(c_{q}-c)}^{T}$ (2)

$W_{k}=\sum_{q=1}^{k} \sum_{x\in A_{q}} (x-c_{q}){(x-c_{q})}^{T}$ (3)

$A_{q}$ is the set of all objects in cluster $q$, $c_{q}$ is the center point of cluster $q$.$n_{q}$ is the number of objects in cluster $q$ and $c$ is the average center of all cluster centers $\{c_{q}\}$.

As shown in Figure S1A, the more clusters we have, the worse clustering effect we have. The elbow method is one of the most popular methods to determine the optimal k-value (number of clusters) for k-means^1^. As the number of clusters increase, the elbow method aims to find a point on which the decrease of cost function reaches a plateau^2^. Fig S1 shows the values for the CHS for the k-means clustering algorithm, using the Elbow criterion. The potential numbers of clusters are those values of k for which the angles are formed. There are elbows for the following values of k: k = 11, k = 17 and k = 21. We set the maximum number of clusters to 25 because there are elbows in it.

The cluster number suggested by elbow method is a subjective result because it is a visual method^3^, and does not provide a measurement metric to show which elbow point is the optimum explicitly. To overcome these shortcomings of the elbow method, two more quantitative discriminant criterion, including DBI and SC, were further applied to determine a value as the estimated potential optimal cluster number for the analyzed dataset.

DBI is an internal evaluation scheme, where the validation of how well the clustering has been done is made using quantities and features inherent to the data set. DBI can characterize the classification appropriateness. The smaller the value of the DBI is, the better the cluster is. Its calculation formula is as follows:

$DBI=\frac{1}{n}\sum_{i=1}^{n} \max_{j\neq i}\left( \frac{\sigma_{i}+\sigma_{j}}{ⅆ\left( c_{i},c_{j} \right)} \right)$ (4)

where n is the number of clusters and$c_{i}$ is thecenter of the $i$-th cluster, and$\sigma_{i}$is the average distance from the center for all objects in cluster $i$, and$ⅆ\left( c_{i},c_{j} \right)$ is the distance between the center points $c_{i}$and$c_{j}$.

SC is a measure of how similar an object is to its own cluster compared to other clusters. The value of SC can indicate the overall effect of clustering. A high value of SC indicates that the object is well matched to its own cluster and poorly matched to neighboring clusters. If most objects have a high value, then the clustering configuration is appropriate. The SC is calculated as follows:

$SC= \frac{1}{N}\sum_{i=1}^{N} \frac{\bar{d_{i}}-ⅆ_{i0}}{\max\left( \bar{d_{i}},d_{i0} \right)}$ (5)

N is the total number of objects and$ⅆ_{i0}$is the average distance of object$i$ from other objects in the same cluster ,and$\bar{d_{i}}$is the average distance of objects in different cluster with the closest distance to object $i$.

Finally we combined the DBI and SC to generate one criterion to determine the most appropriate number of clusters. The DBI and SC for each number of clusters are sorted, and ranks for each sorted list are generated. Then we added up the ranks for each number of clusters and generated the overall ranking index. The smaller the value of the overall ranking index is, the better the cluster is.

**
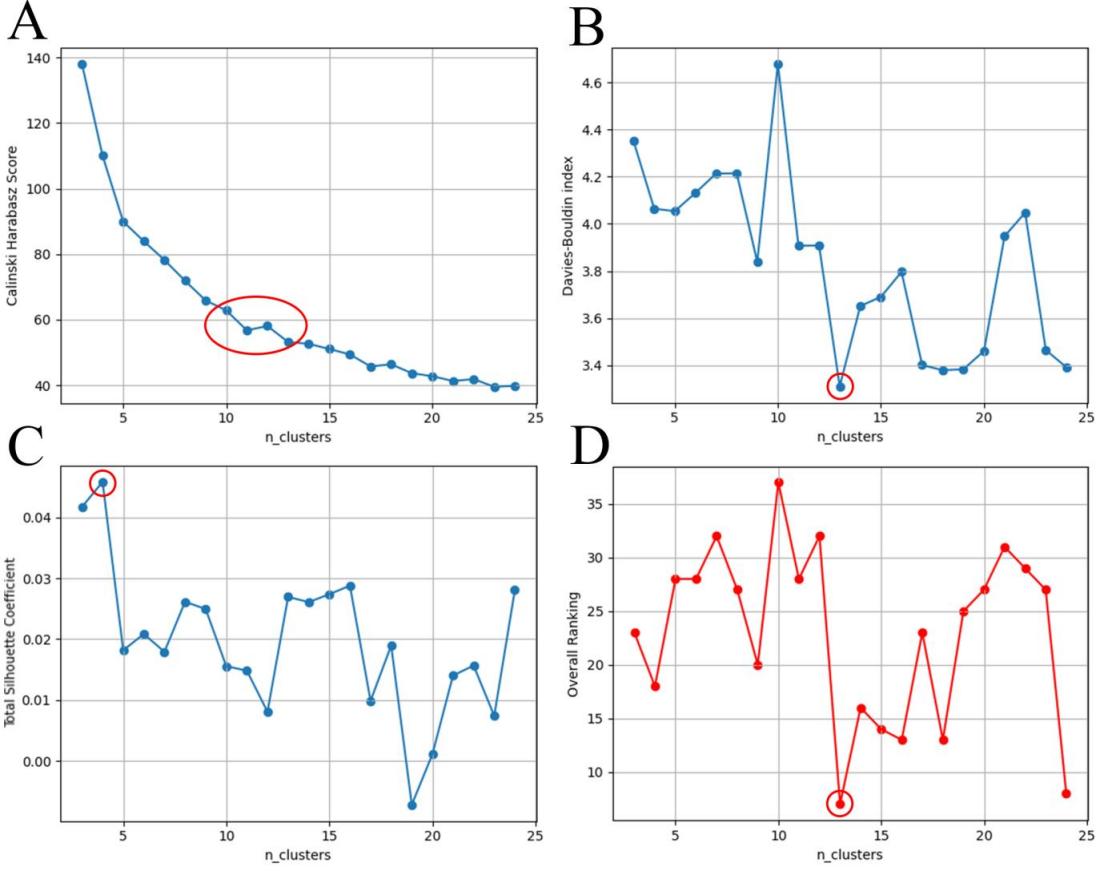
**

**Fig S1. Statistics of data clustering results.** The horizontal axes are the number of clusters, and the vertical axes are the values of K-means clustering evaluation metrics. (A) the Calinski Harabasz Score decreases as the number of clusters increases, red circle represent the first elbow of the curve. (B) A minimum Davies-Bouldin index can be obtained when the number of clusters is 13, which is highlighted by a red circle. (C) A maximum Silhouette Coefficient can be obtained when the number of clusters is 4, which is highlighted by a red circle. (D) A minimum overall ranking index can be obtained when the number of clusters is 13, which is highlighted by a red circle.

**
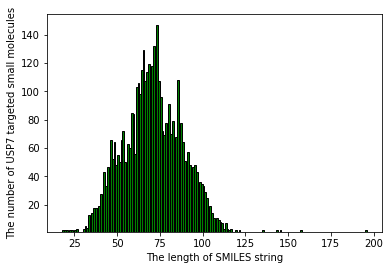
**

**Fig S2. Histogram showing the length of SMILES string in the USP7 targeted small molecules.** The x-axis shows the length of SMILES string and the y-axis shows the corresponding number of USP7 targeted small molecules.

**The reason why the dimensionality of the physicochemical descriptors, MACCS and ECFP4 was reduced to 128 and not any other number.**

The original dimensions of physicochemical descriptors, MACCS and ECFP4 are 196, 166 and 2048, respectively. Based on findings of previous studies, feature extraction yielded better results than applying machine learning directly to the raw data^4, 5^. To improve the model performance and increase the computational efficiency, we used principal component analysis to extract features from physicochemical descriptors, MACCS and ECFP4, and reduce their dimensionality to 128, respectively. The number of dimensions should be a power of 2 to take full advantage of the GPUs processing. Therefore, 32, 64 and 128 were considered. Theoretically, if we set the number of dimension to 128, it will minimize information loss.

In addition, we conducted several pre-experiments to explore whether dimensionality reduction can improve the model performance, and to select the most appropriate number of dimension. Random Forest (RF) classifier, as a widely used classification algorithm, was selected as a representative of nine machine learning classifiers. The deep learning model in Fig.1B in the manuscript was also tested. The physicochemical descriptors, MACCS and ECFP4 were concatenated and used as input to these two models. As shown in Table 1 below, dimensionality reduction can improve the performance of RF, and the most appropriate number of dimension is 128. However, dimensionality reduction would decrease the performance of the DL model in Table 2.

Thus, we reduced the dimensionality of physicochemical descriptors, MACCS and ECFP4 to 128 and not any other number in machine learning experiments. And we didn’t use dimensionality reduction in the deep learning experiments.

**Table S1** The evaluation metrics of the RF classifiers on test set.

| Experiment no. | The number of dimensions | ACC | F1 | AUC |
| --- | --- | --- | --- | --- |
| 1 | 128 | 91.15 | 91.77 | 91.78 |
| 2 | 64 | 84.93 | 85.24 | 86.20 |
| 3 | 32 | 87.57 | 88.21 | 88.42 |
| 4 | Without dimension reduction process | 71.75 | 67.53 | 74.83 |

**Table S2** The evaluation metrics of the DL model on test set.

| Experiment no. | The number of dimensions | ACC | F1 | AUC |
| --- | --- | --- | --- | --- |
| 1 | 128 | 63.65 | 68.92 | 62.50 |
| 2 | 64 | 50.66 | 53.38 | 50.79 |
| 3 | 32 | 56.12 | 66.67 | 52.83 |
| 4 | Without dimension reduction process | 79.28 | 78.17 | 81.42 |

**Table S3** Hyperparameters of ML models considered in optimization

| **Models** | **Hyperparameter distributions** |
| --- | --- |
| SVC | ‘C’: [0.1, 1, 10], ‘kernel’: [‘rbf’, ‘linear’, ‘poly’], ‘gamma’: [1e-3, 1e-4] |
| RF | ‘n_estimators’: [50, 100, 150], ‘criterion’:[‘gini’, ‘entropy’] |
| KNN | ‘weights’: [‘uniform’], ‘n_neighbors’:[4,5,6,7]  ‘weights’: [‘distance’], ‘n_neighbors’:[4,5,6,7], ‘p’:[2,3,4] |
| DT | ‘criterion’:[‘entropy’, ‘gini’], ‘max_depth’:[50,60,100] |
| GBDT | ‘criterion’: [‘friedman_mse’, ‘mae’], ‘n_estimators’: [50,100,150] |
| ABDT | ‘n_estimators’: [50,100,150], ‘learning_rate’: [0.01, 0.1, 1] |
| GNB | ‘var_smoothing’: [1e-9,1e-8,1e-7] |
| SGD | ‘loss’: [‘log’, ‘modified_huber’]  ‘penalty’: [‘l2’, ‘l1’,’elasticnet’] |
| LR | ‘max_iter’: [5000,6000,7000], ‘penalty’: [‘l2’] |

**Table S4. Hyperparameter settings of experiments 8-14 in Group VI.** * Hyperparameter ' b ' is the threshold value of the output probability of the neural network.

| **Experiment index** | **Hyperparameter** |
| --- | --- |
| 8 | ' epochs ': 20, ' batch size ': 128, ' learning rate ': 3e-4, ' dropout rate ': 0.5, ' b ':0.262 |
| 9 | ' epochs ': 20, ' batch size ': 128, ' learning rate ': 1e-3, ' dropout rate ': 0.5, ' b ':0.4 |
| 10 | ' epochs ': 20, ' batch size ': 128, ' learning rate ': 6e-4, ' dropout rate ': 0.5, ' b ':0.408  Remove the dropout layer for linear layers W_Q2, W_K2, W_V2 and maccsfc. |
| 11 | ' epochs ': 20, ' batch size ': 128, ' learning rate ': 4e-4, ' dropout rate ': 0.5, ' b ':0.401 |
| 12 | ' epochs ': 20, ' batch size ': 128, ' learning rate ': 5e-4, ' dropout rate ': 0.5, ' b ':0.4 |
| 13 | ' epochs ': 20, ' batch size ': 128, ' learning rate ': 4e-4, ' dropout rate ': 0.5, ' b ':0.403 |
| 14 | ' epochs ': 20, ' batch size ': 128, ' learning rate ': 4e-4, ' dropout rate ': 0.5, ' b ':0.4 |

**Table S5 Hyperparameter settings of experiments 8-14 in Group VII.** * Hyperparameter ' b ' is the threshold value of the output probability of the neural network.

| **Experiment index** | **Hyperparameter** |
| --- | --- |
| 8 | ' epochs ': 20, ' batch size ': 128, ' learning rate ': 4e-4, ' dropout rate ': 0.5, ' b ':0. 604 |
| 9 | ' epochs ': 20, ' batch size ': 128, ' learning rate ': 1e-3, ' dropout rate ': 0.5, ' b ':0.4 |
| 10 | ' epochs ': 20, ' batch size ': 128, ' learning rate ': 5e-4, ' dropout rate ': 0.5, ' b ':0.6  Remove the dropout layer for linear layers W_Q2, W_K2, W_V2 and maccsfc. |
| 11 | ' epochs ': 20, ' batch size ': 128, ' learning rate ': 4e-4, ' dropout rate ': 0.5, ' b ':0.4 |
| 12 | ' epochs ': 20, ' batch size ': 128, ' learning rate ': 6e-4, ' dropout rate ': 0.5, ' b ':0.401 |
| 13 | ' epochs ': 20, ' batch size ': 128, ' learning rate ': 5e-4, ' dropout rate ': 0.5, ' b ':0.403 |
| 14 | ' epochs ': 20, ' batch size ': 128, ' learning rate ': 4e-4, ' dropout rate ': 0.5, ' b ':0.4 |

**Table S6.** **Hyperparameter settings of experiments 8-14 in Group VIII.** *Hyperparameter ' b ' is the threshold value of the output probability of the neural network.

| **Experiment index** | **Hyperparameter** |
| --- | --- |
| 8 | ' epochs ': 20, ' batch size ': 128, ' learning rate ': 1e-3, ' dropout rate ': 0.6, ' b ':0. 481 |
| 9 | ' epochs ': 20, ' batch size ': 128, ' learning rate ': 1e-3, ' dropout rate ': 0.65, ' b ':0.3 |
| 10 | ' epochs ': 20, ' batch size ': 128, ' learning rate ': 9e-4, ' dropout rate ': 0.4, ' b ':0.865 |
| 11 | ' epochs ': 20, ' batch size ': 128, ' learning rate ': 1e-3, ' dropout rate ': 0.5, ' b ':0.201 |
| 12 | ' epochs ': 20, ' batch size ': 128, ' learning rate ': 9e-4, ' dropout rate ': 0.4, ' b ':0.4 |
| 13 | ' epochs ': 20, ' batch size ': 128, ' learning rate ': 1e-3, ' dropout rate ': 0.5, ' b ':0.403 |
| 14 | ' epochs ': 20, ' batch size ': 128, ' learning rate ': 1e-3, ' dropout rate ': 0.5, ' b ':0.35 |

**Table S7.** **Hyperparameter settings of experiments 8-14 in Group IX.** *Hyperparameter ' b ' is the threshold value of the output probability of the neural network.

| **Experiment index** | **Hyperparameter** |
| --- | --- |
| 8 | ' epochs ': 20, ' batch size ': 128, ' learning rate ': 5e-4, ' dropout rate ': 0.5, ' b ':0. 25 |
| 9 | ' epochs ': 20, ' batch size ': 128, ' learning rate ': 4e-4, ' dropout rate ': 0.65, ' b ':0.15 |
| 10 | ' epochs ': 20, ' batch size ': 128, ' learning rate ': 5e-4, ' dropout rate ': 0.5, ' b ':0.35 |
| 11 | ' epochs ': 20, ' batch size ': 128, ' learning rate ': 4e-4, ' dropout rate ': 0.6, ' b ':0.18 |
| 12 | ' epochs ': 20, ' batch size ': 128, ' learning rate ': 6e-4, ' dropout rate ': 0.6, ' b ':0.3 |
| 13 | ' epochs ': 20, ' batch size ': 128, ' learning rate ': 4e-4, ' dropout rate ': 0.65, ' b ':0.182 |
| 14 | ' epochs ': 20, ' batch size ': 128, ' learning rate ': 4e-4, ' dropout rate ': 0.65, ' b ':0.18 |

**Table S8. Hyperparameter settings of experiments 8-14 in Group X.** *Hyperparameter ' b ' is the threshold value of the output probability of the neural network.

| **Experiment index** | **Hyperparameter** |
| --- | --- |
| 8 | ' epochs ': 20, ' batch size ': 128, ' learning rate ': 1e-3, ' dropout rate ': 0.6, ' b ':0. 45 |
| 9 | ' epochs ': 20, ' batch size ': 128, ' learning rate ': 9e-4, ' dropout rate ': 0.65, ' b ':0.3 |
| 10 | ' epochs ': 20, ' batch size ': 128, ' learning rate ': 1e-3, ' dropout rate ': 0.6, ' b ':0.4 |
| 11 | ' epochs ': 20, ' batch size ': 128, ' learning rate ': 9e-4, ' dropout rate ': 0.6, ' b ':0.398 |
| 12 | ' epochs ': 20, ' batch size ': 128, ' learning rate ': 1e-3, ' dropout rate ': 0.6, ' b ':0.4 |
| 13 | ' epochs ': 20, ' batch size ': 128, ' learning rate ': 1e-3, ' dropout rate ': 0.6, ' b ':0.401 |
| 14 | ' epochs ': 20, ' batch size ': 128, ' learning rate ': 1e-3, ' dropout rate ': 0.6, ' b ':0.35 |

**Table S9.** **Hyperparameter settings of experiments 15 in Group XI-XV.** *Hyperparameter ' b ' is the threshold value of the output probability of the neural network.

| **Group index** | **Hyperparameter** |
| --- | --- |
| XI | ' epochs ': 20, ' batch size ': 128, ' learning rate ': 4e-4, ' dropout rate ': 0.5, ' embedding dim ': 100, ' LSTM hidden dim ': 128, ' LSTM layers ': 4, ' b ':0.4 |
| XII | ' epochs ': 20, ' batch size ': 128, ' learning rate ': 4e-4, ' dropout rate ': 0.5, ' embedding dim ': 100, ' LSTM hidden dim ': 128, ' LSTM layers ': 4, ' b ':0.4 |
| XIII | ' epochs ': 20, ' batch size ': 128, ' learning rate ': 5e-4, ' dropout rate ': 0.5, ' embedding dim ': 100, ' LSTM hidden dim ': 128, ' LSTM layers ': 4, ' b ':0.571 |
| XIV | ' epochs ': 20, ' batch size ': 128, ' learning rate ': 4e-4, ' dropout rate ': 0.5, ' embedding dim ': 100, ' LSTM hidden dim ': 128, ' LSTM layers ': 4, ' b ':0.6187 |
| XV | ' epochs ': 20, ' batch size ': 128, ' learning rate ': 5e-4, ' dropout rate ': 0.5, ' embedding dim ': 100, ' LSTM hidden dim ': 128, ' LSTM layers ': 4, ' b ':0.722 |


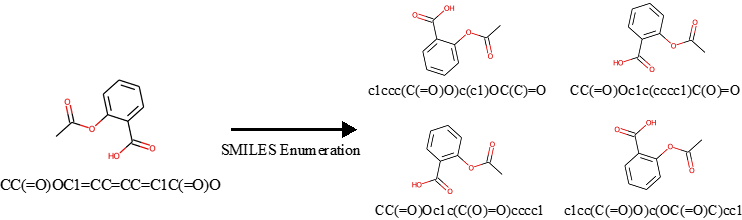


**Figure S2** SMILES enumeration of aspirin, the left is the canonical SMILES

**Evaluation metrics**

Three classification evaluation metrics, including Accuracy (ACC), F1 score and Area Under Receiver Operating Characteristic Curve (AUC), were applied to evaluate the model performance. Two of them are calculated as follows:


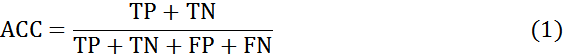


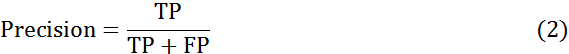


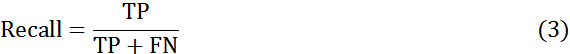


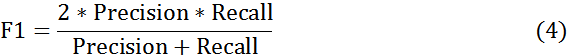


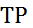
denotes the number of true-positive samples in the test set.
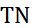
denotes the number of true-negative samples.
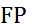
denotes the number of false-positive samples.
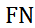
 denotes the number of false-negative samples. The higher ACC reflects a better performance of the model. The recall and precision are applied to evaluate the model’s ability to discriminate positive and negative samples. The higher value of precision indicates the better ability of model to distinguish negative samples. The higher value of recall implies the better ability of model to identify positive samples. F1 score is the harmonic mean of recall and precision, where the higher the value is, the more robust the model will be. AUC measures the ability of the model to predict a higher score for positive examples as compared to negative examples.

**Using t-SNE to Visualize USP7 DataSets**

The physicochemical descriptors, MACCS and ECFP4 of USP7 small molecules were calculated (Methods and Materials). Then these three molecular representations were combined horizontally and normalized using StandardScaler (sklearn.Preprocessing, https://scikit-learn.org). t-SNE was applied to reduce the number of dimensions for USP7 data sets (sklearn.manifold), and PyPlot (matplotlib.pyplot) was used for visualization.

**Table S10.** **t-test results.** If the P value is less than 0.05, a statistically significant difference between two models exists. If the P value is less than 0.01, a statistically extremely significant difference between two models exists. If the P value is greater than 0.05, there is no significant difference between two models.

| **Section**  **Number** | **Models**  **(Group, Exp, Model)** | **P value** | | |
| --- | --- | --- | --- | --- |
|  |  | **ACC** | **F1** | **AUC** |
| 2.1.1 | I, Exp 2, GNB based on ECFP4 | 0 | 0 | 0 |
|  | I, Exp 3, GNB based on MACCS |  |  |  |
| 2.1.1 | I, Exp 2, GNB based on ECFP4 | 0 | 0 | 0 |
|  | I, Exp 1, GNB based on Descriptors |  |  |  |
| 2.1.1 | I, Exp 2, KNN based on ECFP4 | 6.91978E-42 | 5.40665E-14 | 4.52484E-48 |
|  | I, Exp 3, KNN based on MACCS |  |  |  |
| 2.1.1 | I, Exp 2, KNN based on ECFP4 | 5.09654E-45 | 4.91413E-37 | 1.34384E-43 |
|  | I, Exp 1, KNN based on Descriptors |  |  |  |
| 2.1.1 | I, Exp 2, GBDT based on ECFP4 | 3.2555E-49 | 1.775E-43 | 1.37304E-05 |
|  | I, Exp 3, GBDT based on MACCS |  |  |  |
| 2.1.1 | I, Exp 2, GBDT based on ECFP4 | 0 | 2.0232E-191 | 2.74889E-85 |
|  | I, Exp 1, GBDT based on Descriptors |  |  |  |
| 2.1.2 | II, Exp 1, SVC based on Descriptors | 0.164067539 | 1.69271E-19 | 2.85938E-10 |
|  | II, Exp 2, SVC based on ECFP4 |  |  |  |
| 2.1.2 | II, Exp 1, DT based on Descriptors | 7.06431E-38 | 4.87829E-36 | 5.30743E-37 |
|  | II, Exp 2, DT based on ECFP4 |  |  |  |
| 2.1.2 | II, Exp 1, LR based on Descriptors | 0 | 0 | 0 |
|  | II, Exp 2, LR based on ECFP4 |  |  |  |
| 2.1.2 | II, Exp 2, KNN based on ECFP4 | 1.81636E-21 | 1.46929E-05 | 5.50479E-35 |
|  | II, Exp 3, KNN based on MACCS |  |  |  |
| 2.1.2 | II, Exp 2, ABDT based on ECFP4 | 0 | 0 | 0 |
|  | II, Exp 3, ABDT based on MACCS |  |  |  |
| 2.1.2 | II, Exp 2, GNB based on ECFP4 | 0 | 0 | 0 |
|  | II, Exp 3, GNB based on MACCS |  |  |  |
| 2.1.2 | I, Exp1, SVC based on Descriptors | 8.56395E-13 | 6.26932E-18 | 2.64892E-33 |
|  | II, Exp1, SVC based on Descriptors |  |  |  |
| 2.1.2 | I, Exp1, RF based on Descriptors | 7.59645E-08 | 1.62671E-10 | 9.28854E-19 |
|  | II, Exp1, RF based on Descriptors |  |  |  |
| 2.1.2 | I, Exp1, DT based on Descriptors | 1.66994E-18 | 9.54327E-16 | 1.34132E-24 |
|  | II, Exp1, DT based on Descriptors |  |  |  |
| 2.1.2 | I, Exp1, GBDT based on Descriptors | 0 | 0 | 9.18437E-85 |
|  | II, Exp1, GBDT based on Descriptors |  |  |  |
| 2.1.2 | I, Exp1, ABDT based on Descriptors | 0 | 0 | 0 |
|  | II, Exp1, ABDT based on Descriptors |  |  |  |
| 2.1.2 | I, Exp1, GNB based on Descriptors | 0 | 0 | 0 |
|  | II, Exp1, GNB based on Descriptors |  |  |  |
| 2.1.2 | I, Exp1, SGD based on Descriptors | 9.32268E-08 | 1.82572E-08 | 4.62427E-06 |
|  | II, Exp1, SGD based on Descriptors |  |  |  |
| 2.1.2 | I, Exp1, LR based on Descriptors | 0 | 0 | 0 |
|  | II, Exp1, LR based on Descriptors |  |  |  |
| 2.1.2 | II, Exp 1, KNN based on Descriptors | 3.66116E-40 | 2.50812E-41 | 2.03636E-42 |
|  | I, Exp 1, KNN based on Descriptors |  |  |  |
| 2.1.2 | II, Exp 1, DT based on Descriptors | 0.008941782 | 0.546221542 | 3.09045E-38 |
|  | I, Exp 1, GBDT based on Descriptors |  |  |  |
| 2.1.2 | I, Exp2, SVC based on ECFP4 | 7.51927E-22 | 5.60524E-48 | 0.418348585 |
|  | II, Exp2, SVC based on ECFP4 |  |  |  |
| 2.1.2 | I, Exp2, RF based on ECFP4 | 6.93168E-16 | 3.94615E-13 | 5.34435E-06 |
|  | II, Exp2, RF based on ECFP4 |  |  |  |
| 2.1.2 | I, Exp2, DT based on ECFP4 | 2.84607E-20 | 2.29785E-26 | 1.61846E-10 |
|  | II, Exp2, DT based on ECFP4 |  |  |  |
| 2.1.2 | I, Exp2, GBDT based on ECFP4 | 5.57571E-78 | 5.67479E-74 | 3.36534E-55 |
|  | II, Exp2, GBDT based on ECFP4 |  |  |  |
| 2.1.2 | I, Exp2, ABDT based on ECFP4 | 0 | 0 | 0 |
|  | II, Exp2, ABDT based on ECFP4 |  |  |  |
| 2.1.2 | I, Exp2, GNB based on ECFP4 | 0 | 0 | 0 |
|  | II, Exp2, GNB based on ECFP4 |  |  |  |
| 2.1.2 | I, Exp2, SGD based on ECFP4 | 0.000230382 | 0.010801319 | 0.881196549 |
|  | II, Exp2, SGD based on ECFP4 |  |  |  |
| 2.1.2 | I, Exp2, LR based on ECFP4 | 0 | 0 | 0 |
|  | II, Exp2, LR based on ECFP4 |  |  |  |
| 2.1.2 | II, Exp 2, KNN based on ECFP4 | 0.013943165 | 5.82778E-15 | 0.600813832 |
|  | I, Exp 2, KNN based on ECFP4 |  |  |  |
| 2.1.2 | II, Exp 3, SVC based on MACCS | 2.12178E-07 | 0.000335394 | 1.0765E-16 |
|  | I, Exp 3, SVC based on MACCS |  |  |  |
| 2.1.2 | II, Exp 3, KNN based on MACCS | 8.17664E-18 | 1.15466E-14 | 0.45822963 |
|  | I, Exp 3, KNN based on MACCS |  |  |  |
| 2.1.2 | II, Exp 3, SGD based on MACCS | 0.773404156 | 0.170459002 | 0.892318984 |
|  | I, Exp 3, SGD based on MACCS |  |  |  |
| 2.1.2 | II, Exp 3, RF based on MACCS | 6.96467E-37 | 1.81122E-33 | 6.29529E-24 |
|  | I, Exp 3, RF based on MACCS |  |  |  |
| 2.1.2 | II, Exp 3, DT based on MACCS | 2.83629E-08 | 7.75562E-17 | 4.11375E-09 |
|  | I, Exp 3, DT based on MACCS |  |  |  |
| 2.1.2 | II, Exp 3, GBDT based on MACCS | 1.0672E-111 | 3.1492E-116 | 1.2985E-99 |
|  | I, Exp 3, GBDT based on MACCS |  |  |  |
| 2.1.2 | II, Exp 3, ABDT based on MACCS | 0 | 0 | 0 |
|  | I, Exp 3, ABDT based on MACCS |  |  |  |
| 2.1.2 | II, Exp 3, GNB based on MACCS | 0 | 0 | 0 |
|  | I, Exp 3, GNB based on MACCS |  |  |  |
| 2.1.2 | II, Exp 3, LR based on MACCS | 0 | 0 | 0 |
|  | I, Exp 3, LR based on MACCS |  |  |  |
| 2.1.2 | II, Exp 3, ABDT based on ECFP4 | 3.06654E-66 | 5.57789E-64 | 2.08347E-09 |
|  | II, Exp 3, SVC based on MACCS |  |  |  |
| 2.1.3 | III, Exp 2, LR based on ECFP4 | 0 | 0 | 0 |
|  | II, Exp 2, LR based on ECFP4 |  |  |  |
| 2.1.3 | III, Exp 2, LR based on ECFP4 | 0 | 0 | 0 |
|  | I, Exp 2, LR based on ECFP4 |  |  |  |
| 2.1.3 | III, Exp 1, SGD based on Descriptors | 1.35136E-12 | 4.33803E-15 | 0.059941393 |
|  | III, Exp 2, SGD based on ECFP4 |  |  |  |
| 2.1.3 | III, Exp 1, LR based on Descriptors | 0 | 0 | 0 |
|  | III, Exp 2, LR based on ECFP4 |  |  |  |
| 2.1.3 | III, Exp 1, RF based on Descriptors | 3.62999E-28 | 4.65099E-32 | 9.00649E-18 |
|  | I, Exp 1, RF based on Descriptors |  |  |  |
| 2.1.3 | III, Exp 1, RF based on Descriptors | 3.6366E-18 | 1.60266E-28 | 6.66126E-39 |
|  | II, Exp 1, RF based on Descriptors |  |  |  |
| 2.1.3 | III, Exp 1, ABDT based on Descriptors | 0 | 0 | 0 |
|  | I, Exp 1, ABDT based on Descriptors |  |  |  |
| 2.1.3 | III, Exp 1, ABDT based on Descriptors | 0 | 0 | 0 |
|  | II, Exp 1, ABDT based on Descriptors |  |  |  |
| 2.1.3 | III, Exp 1, GBDT based on Descriptors | 1.06459E-57 | 2.2716E-113 | 3.78388E-97 |
|  | I, Exp 1, GBDT based on Descriptors |  |  |  |
| 2.1.3 | III, Exp 1, GBDT based on Descriptors | 1.2888E-129 | 9.6209E-151 | 1.14336E-94 |
|  | II, Exp 1, GBDT based on Descriptors |  |  |  |
| 2.1.3 | III, Exp 2, RF based on ECFP4 | 9.79592E-17 | 0.124574816 | 1.58172E-27 |
|  | I, Exp 2, RF based on ECFP4 |  |  |  |
| 2.1.3 | III, Exp 2, RF based on ECFP4 | 7.72278E-07 | 3.98163E-13 | 1.64763E-22 |
|  | II, Exp 2, RF based on ECFP4 |  |  |  |
| 2.1.3 | III, Exp 2, ABDT based on ECFP4 | 0 | 0 | 0 |
|  | I, Exp 2, ABDT based on ECFP4 |  |  |  |
| 2.1.3 | III, Exp 2, ABDT based on ECFP4 | 0 | 0 | 0 |
|  | II, Exp 2, ABDT based on ECFP4 |  |  |  |
| 2.1.3 | III, Exp 2, GBDT based on ECFP4 | 3.08848E-50 | 2.64962E-48 | 1.94365E-35 |
|  | I, Exp 2, GBDT based on ECFP4 |  |  |  |
| 2.1.3 | III, Exp 2, GBDT based on ECFP4 | 0.965247057 | 1.00977E-34 | 5.51369E-28 |
|  | II, Exp 2, GBDT based on ECFP4 |  |  |  |
| 2.1.3 | III, Exp 3, RF based on MACCS | 1.32272E-51 | 9.19273E-26 | 5.7134E-61 |
|  | I, Exp 3, RF based on MACCS |  |  |  |
| 2.1.3 | III, Exp 3, RF based on MACCS | 0.046112443 | 1.26221E-27 | 1.36249E-52 |
|  | II, Exp 3, RF based on MACCS |  |  |  |
| 2.1.3 | III, Exp 3, ABDT based on MACCS | 0 | 0 | 0 |
|  | I, Exp 3, ABDT based on MACCS |  |  |  |
| 2.1.3 | III, Exp 3, ABDT based on MACCS | 0 | 0 | 0 |
|  | II, Exp 3, ABDT based on MACCS |  |  |  |
| 2.1.3 | III, Exp 3, GBDT based on MACCS | 2.3436E-110 | 2.0312E-101 | 7.5075E-131 |
|  | I, Exp 3, GBDT based on MACCS |  |  |  |
| 2.1.3 | III, Exp 3, GBDT based on MACCS | 4.642E-102 | 1.29162E-81 | 1.3039E-120 |
|  | II, Exp 3, GBDT based on MACCS |  |  |  |
| 2.1.3 | III, Exp 2, GNB based on ECFP4 | 1.77421E-10 | 7.24284E-36 | 4.0578E-21 |
|  | III, Exp 2, RF based on ECFP4 |  |  |  |
| 2.1.3 | III, Exp 2, RF based on ECFP4 | 1.33259E-42 | 2.21993E-37 | 2.98987E-28 |
|  | I, Exp 3, ABDT based on MACCS |  |  |  |
| 2.1.3 | III, Exp 2, RF based on ECFP4 | 1.37487E-31 | 1.1591E-09 | 1.32491E-19 |
|  | II, Exp 2, ABDT based on ECFP4 |  |  |  |
| 2.2.1 | I, Exp 7, GBDT based on Descriptors+ECFP4+ MACCS | 0 | 0 | 2.72301E-35 |
|  | I, Exp 3, ABDT based on MACCS |  |  |  |
| 2.2.2 | II, Exp 7, GBDT based on Descriptors+ECFP4+ MACCS | 4.20054E-54 | 2.71029E-55 | 2.07327E-27 |
|  | II, Exp 2, ABDT based on ECFP4 |  |  |  |
| 2.2.3 | III, Exp 4, RF based on Descriptors+ECFP4 | 0.009213659 | 0.041123128 | 0.003008206 |
|  | III, Exp 2, RF based on ECFP4 |  |  |  |
|  | V, Exp 3, LR based on MACCS + Decoy |  |  |  |
| 2.2.3 | IV, Exp 4, RF based on Descriptors+  ECFP4+SMOTE | 0.00712352 | 0.05011496 | 0.079053817 |
|  | IV, Exp 2, RF based on ECFP4+SMOTE |  |  |  |
| 3.1.2 | VII, Exp 14, DL based on Descriptors+ MACCS+ECFP4 | 2.91323E-11 | 2.22585E-09 | 0.001126297 |
|  | VI, Exp 13, DL based on MACCS+ECFP4 |  |  |  |
| 3.1.3 | VIII, Exp 13, DL based on MACCS+ECFP4 | 6.55346E-22 | 5.35564E-15 | 3.13224E-28 |
|  | VI, Exp 13, DL based on MACCS+ECFP4 |  |  |  |
| 3.1.3 | VIII, Exp 13, DL based on MACCS+ECFP4 | 3.24173E-15 | 6.91456E-09 | 1.51939E-25 |
|  | VII, Exp 14, DL based on Descriptors+ MACCS+ECFP4 |  |  |  |
| 3.1.3 | IX, Exp 12, DL  based on Descriptors+  MACCS+SMOTE | 3.9737E-09 | 2.4198E-08 | 0.000202499 |
|  | VIII, Exp 13, DL based on MACCS+ECFP4 |  |  |  |
| 3.1.3 | X, Exp 11, DL based on Descriptors+ECFP4  +Decoy | 7.51935E-11 | 2.03973E-09 | 0.002331919 |
|  | VIII, Exp 13, DL based on MACCS+ECFP4 |  |  |  |
| 3.2 | XIV, Exp 15, DL based on SMILES | 0.095167248 | 1.33714E-07 | 4.89378E-09 |
|  | XV, Exp 15, DL based on SMILES |  |  |  |
| 3.2 | XV, Exp 15, DL based on SMILES | 8.64217E-06 | 0.495413363 | 0.002104267 |
|  | XIII, Exp 15, DL based on SMILES |  |  |  |
| 3.2 | XIV, Exp 15, DL based on SMILES | 5.07609E-06 | 1.75292E-06 | 0.000627297 |
|  | XIII, Exp 15, DL based on SMILES |  |  |  |
| 4 | I, Exp 3, ABDT based on MACCS | 0 | 0 | 0 |
|  | I, Exp 4, ABDT based on Descriptors+ECFP4  Or  I, Exp 7, ABDT based on Descriptors+ECFP4+MACCS |  |  |  |
| 4 | I, Exp 3, ABDT based on MACCS | 0 | 0 | 0 |
|  | I, Exp 4, GBDT based on Descriptors+ECFP4 |  |  |  |
| 4 | I, Exp 3, ABDT based on MACCS | 0 | 0 | 2.72301E-35 |
|  | I, Exp 7, GBDT based on Descriptors+ECFP4+MACCS |  |  |  |
| 4 | VI, Exp 13, DL based on MACCS+ECFP4 (rank 1) | 0.003781141 | 0.135681959 | 3.0267E-07 |
|  | VI, Exp 8, DL based on Descriptors (rank 4) |  |  |  |
| 4 | VI, Exp 13, DL based on MACCS+ECFP4 (rank 1) | 0.048825127 | 0.132157191 | 9.39614E-07 |
|  | VI, Exp 10, DL based on MACCS (rank 3) |  |  |  |
| 4 | VI, Exp 13, DL based on MACCS+ECFP4 (rank 1) | 0.062031 | 0.166976 | 0.35266 |
|  | VI, Exp 12, DL based on Descriptors+MACCS (rank 2) |  |  |  |
| 4 | II, Exp 7, GBDT based on Descriptors+ECFP4+MACCS | 4.20054E-54 | 2.71029E-55 | 2.07327E-27 |
|  | II, Exp 2, ABDT based on ECFP4 |  |  |  |
| 4 | II, Exp 7, GBDT based on Descriptors+ECFP4+MACCS | 1.16541E-06 | 2.16754E-06 | 7.97212E-48 |
|  | II, Exp 4, GBDT based on Descriptors+ECFP4 |  |  |  |
| 4 | II, Exp 7, GBDT based on Descriptors+ECFP4+MACCS | 6.1137E-36 | 8.68079E-33 | 1.36007E-64 |
|  | II, Exp 5, SVC based on Descriptors +MACCS |  |  |  |
| 4 | VII, Exp 14, DL based on Descriptors +MACCS+ECFP4 (rank 1) | 0.006696161 | 0.004753787 | 0.52864264 |
|  | VII, Exp 11, DL based on Descriptors+ECFP4 (rank 3) |  |  |  |
| 4 | VII, Exp 14, DL based on Descriptors +MACCS+ECFP4 (rank 1) | 0.002449401 | 0.000889647 | 0.448478973 |
|  | VII, Exp 12, DL based on Descriptors +MACCS (rank 4) |  |  |  |
| 4 | VII, Exp 14, DL based on Descriptors +MACCS+ECFP4 (rank 1) | 0.110304486 | 0.124071736 | 0.937241265 |
|  | VII, Exp 13, DL based on MACCS+ECFP4 (rank 2) |  |  |  |
| 4 | IV, Exp 2, RF based on ECFP4+SMOTE | 1.83807E-28 | 1.48987E-36 | 0.883843757 |
|  | IV, Exp 2, GNB based on ECFP4+SMOTE |  |  |  |
| 4 | IV, Exp 2, RF based on ECFP4+SMOTE | 0.00712352 | 0.05011496 | 0.079053817 |
|  | IV, Exp 4, RF based on Descriptors+ECFP4+SMOTE |  |  |  |
| 4 | IV, Exp 2, RF based on ECFP4+SMOTE | 3.32864E-28 | 5.61301E-36 | 3.20928E-05 |
|  | IV, Exp 4, GBDT based on Descriptors+ECFP4+SMOTE |  |  |  |
| 4 | IX, Exp 12, DL based on Descriptors +MACCS+SMOTE (rank 1) | 0.120330118 | 0.159978378 | 0.46674147 |
|  | IX, Exp 10, DL based on MACCS+SMOTE (rank 4) |  |  |  |
| 4 | IX, Exp 12, DL based on Descriptors +MACCS+SMOTE (rank 1) | 0.232199567 | 0.0541116 | 0.689477173 |
|  | IX, Exp 13, DL based on ECFP4 +MACCS+SMOTE (rank 2) |  |  |  |
| 4 | IX, Exp 12, DL based on Descriptors +MACCS+SMOTE (rank 1) | 0.005184428 | 7.47115E-05 | 0.643037954 |
|  | IX, Exp 14, DL based on Descriptors +MACCS+ECFP4+SMOTE (rank 3) |  |  |  |
| 4 | IX, Exp 12, DL based on Descriptors +MACCS+SMOTE (rank 1) | 0.100647892 | 0.161232242 | 0.054951898 |
|  | X, Exp 11, DL based on Descriptors+ECFP4+Decoy (rank 5) |  |  |  |
| 4 | IX, Exp 12, DL based on Descriptors +MACCS+SMOTE (rank 1) | 0.574250752 | 0.892433662 | 0.000467427 |
|  | X, Exp 14, DL based on Descriptors +ECFP4+MACCS+Decoy (rank 6) |  |  |  |
| 4 | IX, Exp 12, DL based on Descriptors +MACCS+SMOTE (rank 1) | 0.317104592 | 0.31242743 | 0.00014314 |
|  | X, Exp 9, DL based on ECFP4+Decoy (rank 7) |  |  |  |
| 4 | IX, Exp 12, DL based on Descriptors +MACCS+SMOTE (rank 1) | 0.215303255 | 0.10225547 | 2.63862E-05 |
|  | X, Exp 13, DL based on ECFP4+MACCS+Decoy (rank 8) |  |  |  |
| 4 | I, Exp 3, ABDT based on MACCS | 7.01264E-19 | 2.52365E-17 | 1.95766E-11 |
|  | VI, Exp 13, MACCS+ECFP4 (rank 1) |  |  |  |
| 4 | I, Exp 3, ABDT based on MACCS | 4.45892E-21 | 8.43424E-19 | 2.03772E-05 |
|  | VI, Exp 12, DL based on Physicochemical descriptors +MACCS (rank 2) |  |  |  |
| 4 | I, Exp 3, ABDT based on MACCS | 1.14284E-15 | 1.08594E-12 | 9.36742E-23 |
|  | XI, Exp15, DL based on SMILES |  |  |  |
| 4 | XII, Exp15, DL based on SMILES | 5.61992E-06 | 1.15992E-07 | 1.39244E-22 |
|  | II, Exp 7, GBDT based on Descriptors+ECFP4+MACCS |  |  |  |
| 4 | XII, Exp15, DL based on SMILES | 1.72369E-13 | 4.34715E-13 | 0.012194454 |
|  | VII, Exp 14, DL based on Descriptors +MACCS+ECFP4 |  |  |  |
| 4 | XII, Exp15, DL based on SMILES | 8.88729E-10 | 6.39239E-10 | 0.01788387 |
|  | VII, Exp 13, DL based on MACCS+ECFP4 |  |  |  |
| 4 | IV, Exp 2, RF based on ECFP4+SMOTE | 5.63881E-22 | 5.6804E-23 | 8.87998E-14 |
|  | IX, Exp 12, DL based on Descriptors+MACCS +SMOTE |  |  |  |
| 4 | IV, Exp 2, RF based on ECFP4+SMOTE | 2.05851E-25 | 1.72433E-30 | 2.00419E-33 |
|  | XIV, Exp15, DL based on SMILES+SMILES enumeration |  |  |  |
| 4 | IV, Exp 2, RF based on ECFP4+SMOTE | 1.71107E-15 | 1.61813E-15 | 1.22006E-15 |
|  | IX, Exp 13, DL based on ECFP4+MACCS +SMOTE |  |  |  |
| 4 | IV, Exp 2, RF based on ECFP4+SMOTE | 3.01347E-28 | 1.45366E-31 | 3.04138E-14 |
|  | IX, Exp 10, DL based on MACCS+SMOTE |  |  |  |
| 4 | IV, Exp 2, RF based on ECFP4+SMOTE | 2.3407E-32 | 6.32241E-36 | 4.97135E-31 |
|  | X, Exp 11, DL based on Descriptors+ECFP4+Decoy |  |  |  |
| 4 | XI, Exp15, DL based on SMILES | 2.99546E-08 | 3.02289E-09 | 0.023016973 |
|  | VI, Exp 13, DL based on MACCS+ECFP4 |  |  |  |
| 4 | XI, Exp15, DL based on SMILES | 1.79518E-11 | 1.95102E-11 | 0.006706639 |
|  | VI, Exp 12, DL based on Descriptors+MACCS |  |  |  |

**Table S11. The evaluation metrics of the ML models on test set in Groups I-II (mean ± SD, %).** * Exp means the experiment no. in each group.

| Exp | Group | Metrics | SVC | RF | KNN | DT | GBDT | ABDT | GNB | SGD | LR |
| --- | --- | --- | --- | --- | --- | --- | --- | --- | --- | --- | --- |
| **1** | Ⅰ | ACC | 52.17±2.15 | 59.55±4.18 | 62.55±0.58 | 66.42±3.48 | 76.27±0.00 | 65.73±0.00 | 70.62±0.00 | 44.55±2.38 | 43.50±0.00 |
|  |  | F1 | 28.94±6.62 | 44.91±9.21 | 70.77±0.31 | 65.44±4.89 | 75.77±0.00 | 57.87±0.00 | 75.32±0.00 | 17.01±5.72 | 12.79±0.00 |
|  |  | AUC | 85.34±1.06 | 83.07±3.27 | 68.52±0.67 | 67.94±3.12 | 87.30±0.00 | 84.25±0.00 | 77.36±0.00 | 67.11±7.87 | 71.87±0.00 |
|  | Ⅱ | ACC | 55.74±0.00 | 66.04±3.99 | 70.42±1.12 | 75.44±1.68 | 56.31±0.00 | 67.98±0.00 | 67.80±0.00 | 51.31±5.58 | 52.35±0.00 |
|  |  | F1 | 43.91±0.00 | 60.89±6.55 | 76.83±0.87 | 75.99±1.96 | 45.79±0.00 | 64.73±0.00 | 74.05±0.00 | 33.26±12.38 | 37.22±0.00 |
|  |  | AUC | 90.27±0.00 | 91.01±0.79 | 75.95±0.85 | 78.85±1.47 | 86.95±0.01 | 90.98±0.00 | 73.11±0.00 | 80.77±12.53 | 88.40±0.00 |
| **2** | Ⅰ | ACC | 66.25±3.81 | 87.28±4.27 | 70.03±0.79 | 70.24±4.23 | 91.90±0.00 | 92.09±0.00 | 89.83±0.00 | 57.60±3.04 | 56.87±0.00 |
|  |  | F1 | 72.76±3.48 | 87.60±4.95 | 75.21±0.75 | 72.27±5.29 | 92.60±0.01 | 92.78±0.00 | 90.49±0.00 | 43.21±5.66 | 45.35±0.00 |
|  |  | AUC | 75.30±5.73 | 94.09±0.75 | 76.47±0.88 | 70.52±3.89 | 93.07±0.15 | 92.46±0.00 | 93.44±0.00 | 75.73±9.50 | 72.02±0.00 |
|  | Ⅱ | ACC | 55.65±0.37 | 68.39±8.34 | 70.65±1.09 | 57.41±2.69 | 77.79±0.49 | 86.63±0.00 | 74.20±0.00 | 53.58±4.71 | 50.09±0.00 |
|  |  | F1 | 42.06±0.75 | 63.90±13.02 | 77.04±0.60 | 46.80±5.20 | 77.95±0.60 | 87.78±0.00 | 79.88±0.00 | 37.31±10.89 | 29.33±0.00 |
|  |  | AUC | 76.97±9.59 | 92.78±1.22 | 76.37±0.64 | 64.16±2.25 | 90.87±0.12 | 91.02±0.00 | 85.57±0.00 | 76.16±12.65 | 69.13±0.00 |
| **3** | Ⅰ | ACC | 69.00±10.20 | 89.20±1.35 | 63.43±0.59 | 73.04±0.78 | 91.31±0.07 | 92.28±0.00 | 71.37±0.00 | 70.29±5.51 | 75.33±0.00 |
|  |  | F1 | 77.41±4.32 | 89.77±1.48 | 73.70±0.38 | 74.17±0.87 | 92.21±0.05 | 93.02±0.00 | 72.16±0.00 | 65.41±8.87 | 73.10±0.00 |
|  |  | AUC | 80.02±4.39 | 94.31±0.45 | 63.93±1.15 | 73.92±0.76 | 92.94±0.03 | 93.94±0.00 | 79.13±0.00 | 85.96±8.49 | 91.56±0.00 |
|  | Ⅱ | ACC | 79.96±0.23 | 72.81±2.69 | 66.34±1.15 | 71.05±1.51 | 84.18±0.00 | 79.28±0.00 | 66.48±0.00 | 70.65±3.95 | 74.01±0.00 |
|  |  | F1 | 80.42±0.29 | 70.93±3.72 | 75.94±1.13 | 69.30±2.11 | 85.16±0.00 | 79.55±0.00 | 67.28±0.00 | 68.06±5.55 | 72.94±0.00 |
|  |  | AUC | 89.30±0.06 | 90.08±1.30 | 63.56±2.47 | 75.76±1.24 | 88.45±0.06 | 94.10±0.00 | 76.31±0.00 | 85.70±5.76 | 88.97±0.00 |
| **4** | Ⅰ | ACC | 71.11±1.11 | 83.77±4.75 | 66.55±0.71 | 63.29±2.63 | 92.09±0.00 | 92.09±0.00 | 77.78±0.00 | 49.48±1.85 | 51.22±0.00 |
|  |  | F1 | 71.36±2.45 | 83.54±5.80 | 72.78±0.61 | 63.22±3.31 | 92.78±0.00 | 92.78±0.00 | 80.66±0.00 | 28.29±4.66 | 39.06±0.00 |
|  |  | AUC | 80.79±1.33 | 94.63±0.84 | 74.18±0.49 | 64.72±2.53 | 93.19±0.00 | 93.61±0.00 | 84.14±0.00 | 59.93±6.82 | 58.47±0.00 |
|  | Ⅱ | ACC | 53.65±4.65 | 68.47±8.34 | 69.77±0.58 | 76.60±7.32 | 86.08±1.61 | 76.27±0.00 | 71.19±0.00 | 51.76±4.39 | 44.82±0.00 |
|  |  | F1 | 38.47±10.23 | 63.92±12.87 | 76.86±0.44 | 75.77±10.30 | 87.27±1.68 | 76.14±0.00 | 77.20±0.00 | 33.94±9.92 | 16.05±0.00 |
|  |  | AUC | 85.62±3.21 | 92.95±1.10 | 73.69±0.38 | 80.04±5.96 | 94.12±0.25 | 90.57±0.00 | 81.54±0.00 | 74.55±14.05 | 58.61±0.00 |
| **5** | Ⅰ | ACC | 75.18±6.03 | 80.95±3.35 | 59.67±0.43 | 68.79±1.29 | 88.28±0.08 | 80.23±0.00 | 73.26±0.00 | 65.32±3.90 | 68.55±0.00 |
|  |  | F1 | 77.76±1.89 | 80.12±4.34 | 70.17±0.20 | 74.37±0.98 | 88.93±0.07 | 79.61±0.00 | 74.91±0.00 | 61.23±6.45 | 67.82±0.00 |
|  |  | AUC | 82.58±3.44 | 94.49±1.00 | 67.64±0.66 | 67.08±1.41 | 93.36±0.02 | 91.44±0.00 | 78.60±0.00 | 78.08±9.09 | 83.33±0.00 |
|  | Ⅱ | ACC | 83.45±0.80 | 74.53±2.60 | 65.73±1.28 | 74.47±1.69 | 82.68±0.03 | 80.98±0.00 | 69.87±0.00 | 59.57±5.14 | 76.27±0.00 |
|  |  | F1 | 84.81±0.89 | 73.26±3.59 | 75.18±1.03 | 74.73±1.98 | 83.52±0.04 | 81.54±0.00 | 73.33±0.00 | 49.75±9.55 | 76.75±0.00 |
|  |  | AUC | 94.46±0.07 | 91.88±0.78 | 66.76±1.14 | 78.60±2.72 | 90.91±0.02 | 91.49±0.00 | 76.83±0.00 | 83.40±10.90 | 92.17±0.00 |
| **6** | Ⅰ | ACC | 78.27±6.20 | 90.62±1.30 | 63.78±0.33 | 54.65±4.77 | 91.53±0.00 | 92.09±0.00 | 76.46±0.00 | 57.41±2.80 | 58.57±0.00 |
|  |  | F1 | 81.15±2.96 | 91.25±1.35 | 72.98±0.13 | 51.98±9.89 | 92.31±0.00 | 92.78±0.00 | 77.56±0.00 | 45.92±5.14 | 48.60±0.00 |
|  |  | AUC | 85.88±2.99 | 94.63±0.71 | 71.61±0.86 | 56.85±3.70 | 92.08±0.03 | 92.46±0.00 | 83.77±0.00 | 73.92±11.63 | 85.28±0.00 |
|  | Ⅱ | ACC | 65.96±0.91 | 79.29±3.40 | 65.88±0.90 | 81.78±9.66 | 69.74±0.11 | 64.60±0.00 | 75.89±0.00 | 84.33±3.07 | 63.28±0.00 |
|  |  | F1 | 61.22±1.75 | 79.36±4.18 | 75.00±0.64 | 81.42±14.85 | 67.25±0.17 | 58.95±0.00 | 78.67±0.00 | 85.18±3.51 | 56.57±0.00 |
|  |  | AUC | 89.21±2.12 | 93.39±1.18 | 67.35±0.27 | 84.33±8.10 | 92.08±0.10 | 89.16±0.00 | 82.05±0.00 | 88.84±1.84 | 93.60±0.00 |
| **7** | Ⅰ | ACC | 79.24±1.20 | 89.55±1.79 | 65.07±0.18 | 75.75±3.81 | 92.09±0.00 | 92.09±0.00 | 75.71±0.00 | 57.42±2.23 | 56.87±0.00 |
|  |  | F1 | 79.93±1.28 | 90.15±1.91 | 72.68±0.07 | 78.09±4.09 | 92.78±0.00 | 92.78±0.00 | 77.49±0.00 | 47.90±4.35 | 49.89±0.00 |
|  |  | AUC | 86.18±0.97 | 95.18±0.70 | 72.97±0.40 | 75.61±3.68 | 93.63±0.06 | 93.61±0.00 | 83.00±0.00 | 71.80±10.65 | 78.24±0.00 |
|  | Ⅱ | ACC | 75.46±2.39 | 77.37±3.25 | 68.33±0.59 | 80.77±9.01 | 87.68±0.09 | 66.10±0.00 | 72.88±0.00 | 82.64±3.89 | 75.52±0.00 |
|  |  | F1 | 75.66±2.02 | 77.04±4.09 | 76.43±0.55 | 80.45±14.16 | 88.88±0.10 | 62.03±0.00 | 77.14±0.00 | 83.68±4.33 | 75.29±0.00 |
|  |  | AUC | 93.77±1.82 | 93.39±0.88 | 71.55±0.34 | 84.49±7.87 | 91.59±0.16 | 90.06±0.00 | 79.96±0.00 | 87.25±3.61 | 91.57±0.00 |

**Table S12. The evaluation metrics of the ML models on test set in Groups III-V (mean ± SD, %).** * Exp means the experiment no. In each group.

| Exp | Group | Metrics | SVC | RF | KNN | DT | GBDT | ABDT | GNB | SGD | LR |
| --- | --- | --- | --- | --- | --- | --- | --- | --- | --- | --- | --- |
| **1** | Ⅲ | ACC | 75.33±0.00 | 75.27±0.56 | 73.19±0.39 | 67.54±6.58 | 77.48±0.09 | 75.33±0.00 | 62.52±0.00 | 74.73±4.33 | 74.01±0.00 |
|  |  | F1 | 85.93±0.00 | 85.72±0.34 | 84.37±0.24 | 76.34±6.10 | 84.56±0.08 | 85.93±0.00 | 74.78±0.00 | 81.23±3.78 | 81.30±0.00 |
|  |  | AUC | 63.52±5.25 | 73.30±2.88 | 53.32±0.85 | 63.62±5.88 | 83.20±0.07 | 82.25±0.00 | 59.33±0.00 | 77.58±3.61 | 73.35±0.00 |
|  | Ⅳ | ACC | 61.89±8.16 | 73.80±1.72 | 66.88±2.16 | 54.39±4.09 | 60.28±0.10 | 62.52±0.00 | 66.10±0.00 | 63.62±10.52 | 52.35±0.00 |
|  |  | F1 | 70.17±9.53 | 84.32±1.23 | 77.59±1.74 | 63.86±3.55 | 66.05±0.12 | 71.37±0.00 | 78.31±0.00 | 68.93±11.32 | 56.15±0.00 |
|  |  | AUC | 63.16±3.54 | 66.22±3.31 | 57.89±2.01 | 55.12±4.55 | 75.03±0.09 | 69.52±0.00 | 56.03±0.00 | 73.80±4.64 | 72.76±0.00 |
|  | Ⅴ | ACC | 67.05±2.84 | 70.09±1.41 | 74.04±0.17 | 57.01±6.28 | 77.24±0.48 | 52.17±0.00 | 58.95±0.00 | 61.92±7.26 | 65.73±0.00 |
|  |  | F1 | 77.46±2.37 | 76.16±1.38 | 84.66±0.15 | 66.80±5.42 | 82.68±0.40 | 59.94±0.00 | 71.54±0.00 | 68.69±7.95 | 73.39±0.00 |
|  |  | AUC | 69.02±3.35 | 76.07±1.12 | 63.47±0.21 | 56.49±6.72 | 81.67±0.60 | 61.86±0.00 | 56.07±0.00 | 70.36±7.27 | 73.64±0.00 |
| **2** | Ⅲ | ACC | 75.33±0.00 | 77.11±2.19 | 74.79±0.69 | 70.73±1.81 | 77.80±1.49 | 75.33±0.00 | 74.01±0.00 | 65.84±3.25 | 71.37±0.00 |
|  |  | F1 | 85.93±0.00 | 86.15±1.23 | 85.22±0.41 | 80.50±1.25 | 83.78±1.01 | 85.93±0.00 | 79.71±0.00 | 71.45±3.40 | 77.04±0.00 |
|  |  | AUC | 64.11±4.44 | 85.36±2.29 | 46.26±7.48 | 61.02±2.32 | 85.20±1.53 | 82.59±0.00 | 79.24±0.00 | 75.90±3.13 | 74.54±0.00 |
|  | Ⅳ | ACC | 72.97±2.38 | 84.94±1.25 | 54.96±5.70 | 74.34±2.64 | 78.87±0.22 | 80.41±0.00 | 80.23±0.00 | 60.69±3.05 | 63.09±0.00 |
|  |  | F1 | 82.86±2.54 | 90.04±0.84 | 65.83±5.74 | 82.89±1.58 | 84.28±0.19 | 85.87±0.00 | 85.52±0.00 | 65.88±4.09 | 68.18±0.00 |
|  |  | AUC | 61.74±2.67 | 90.20±0.97 | 53.18±6.13 | 64.95±4.53 | 88.80±0.11 | 88.13±0.00 | 90.23±0.00 | 73.60±3.16 | 74.26±0.00 |
|  | Ⅴ | ACC | 48.72±0.79 | 75.17±0.62 | 71.64±0.23 | 74.16±2.85 | 75.71±0.00 | 74.95±0.00 | 67.04±0.00 | 50.62±4.58 | 53.86±0.00 |
|  |  | F1 | 48.97±1.22 | 80.55±0.58 | 82.34±0.27 | 79.67±2.81 | 81.06±0.00 | 80.35±0.00 | 77.59±0.00 | 51.39±7.12 | 56.33±0.00 |
|  |  | AUC | 88.68±0.87 | 85.84±1.11 | 59.37±0.58 | 81.01±1.68 | 85.46±0.00 | 83.25±0.00 | 75.29±0.00 | 80.71±8.12 | 85.42±0.00 |
| **3** | Ⅲ | ACC | 75.33±0.00 | 73.84±0.73 | 73.09±0.27 | 76.67±1.61 | 68.54±0.21 | 74.20±0.00 | 59.70±0.00 | 65.78±3.46 | 65.54±0.00 |
|  |  | F1 | 85.93±0.00 | 84.58±0.49 | 84.39±0.18 | 83.99±1.41 | 79.80±0.16 | 85.19±0.00 | 69.08±0.00 | 75.51±2.59 | 76.45±0.00 |
|  |  | AUC | 63.19±3.39 | 66.53±1.85 | 52.11±1.08 | 72.31±1.88 | 73.07±0.08 | 42.11±0.00 | 64.15±0.00 | 64.57±4.49 | 69.08±0.00 |
|  | Ⅳ | ACC | 66.73±2.89 | 72.60±2.64 | 64.79±1.03 | 69.90±1.62 | 72.13±0.43 | 70.43±0.00 | 60.08±0.00 | 62.98±2.85 | 64.22±0.00 |
|  |  | F1 | 78.68±2.00 | 82.95±2.01 | 75.91±0.83 | 81.03±1.24 | 80.94±0.37 | 80.10±0.00 | 68.91±0.00 | 71.94±2.22 | 73.09±0.00 |
|  |  | AUC | 54.77±6.32 | 74.33±2.73 | 58.56±0.74 | 53.63±3.12 | 73.44±0.16 | 61.17±0.00 | 65.93±0.00 | 65.23±3.11 | 67.09±0.00 |
|  | Ⅴ | ACC | 63.81±1.51 | 69.19±0.75 | 71.48±0.37 | 65.48±1.07 | 72.10±0.17 | 69.49±0.00 | 56.50±0.00 | 61.76±3.12 | 64.22±0.00 |
|  |  | F1 | 74.99±0.58 | 74.51±0.81 | 82.97±0.39 | 72.42±1.10 | 77.61±0.17 | 76.32±0.00 | 66.47±0.00 | 69.58±3.84 | 74.32±0.00 |
|  |  | AUC | 69.76±1.40 | 75.76±0.94 | 50.40±0.01 | 70.87±0.99 | 73.81±0.07 | 74.04±0.00 | 61.15±0.00 | 67.83±3.58 | 65.10±0.00 |
| **4** | Ⅲ | ACC | 75.33±0.00 | 75.88±1.20 | 73.26±0.22 | 75.04±1.37 | 78.39±0.78 | 75.33±0.00 | 60.45±0.00 | 72.28±2.62 | 70.06±0.00 |
|  |  | F1 | 85.93±0.00 | 85.61±0.67 | 84.43±0.15 | 82.61±1.01 | 84.00±0.54 | 85.93±0.00 | 72.22±0.00 | 81.99±1.78 | 77.38±0.00 |
|  |  | AUC | 66.79±5.94 | 83.59±2.13 | 48.21±1.93 | 71.17±1.89 | 83.43±0.64 | 82.59±0.00 | 69.94±0.00 | 64.01±6.91 | 78.65±0.00 |
|  | Ⅳ | ACC | 74.03±2.07 | 83.87±1.70 | 60.03±4.65 | 79.94±2.30 | 79.87±0.53 | 77.97±0.00 | 71.56±0.00 | 61.14±5.08 | 62.71±0.00 |
|  |  | F1 | 84.27±2.31 | 89.54±1.06 | 72.45±4.11 | 86.77±1.45 | 85.02±0.46 | 83.36±0.00 | 80.00±0.00 | 72.05±3.99 | 69.44±0.00 |
|  |  | AUC | 61.85±4.25 | 89.66±1.35 | 52.84±4.54 | 71.44±4.65 | 89.40±0.09 | 87.31±0.00 | 77.50±0.00 | 55.90±9.51 | 73.61±0.00 |
|  | Ⅴ | ACC | 72.59±3.76 | 74.48±0.79 | 73.62±0.63 | 63.03±2.45 | 76.08±0.15 | 75.71±0.00 | 63.09±0.00 | 77.06±1.86 | 67.23±0.00 |
|  |  | F1 | 81.75±4.14 | 79.90±0.75 | 84.35±0.46 | 68.82±2.89 | 81.41±0.14 | 81.65±0.00 | 74.87±0.00 | 82.36±1.70 | 72.81±0.00 |
|  |  | AUC | 73.30±3.19 | 83.65±1.35 | 57.18±0.66 | 72.19±1.97 | 85.89±0.16 | 78.08±0.00 | 64.17±0.00 | 82.84±1.26 | 85.48±0.00 |
| **5** | Ⅲ | ACC | 75.33±0.00 | 74.08±0.98 | 73.03±0.11 | 60.26±2.33 | 71.56±0.00 | 75.33±0.00 | 61.58±0.00 | 71.10±2.71 | 72.32±0.00 |
|  |  | F1 | 85.93±0.00 | 84.69±0.72 | 84.28±0.06 | 70.92±2.96 | 80.81±0.00 | 85.93±0.00 | 71.67±0.00 | 81.15±2.03 | 82.40±0.00 |
|  |  | AUC | 66.12±3.20 | 71.69±2.07 | 52.90±0.43 | 55.35±2.72 | 78.52±0.03 | 71.94±0.00 | 61.55±0.00 | 61.10±4.98 | 63.83±0.00 |
|  | Ⅳ | ACC | 74.29±0.89 | 75.08±1.37 | 64.62±0.27 | 58.01±5.49 | 76.14±0.18 | 68.55±0.00 | 61.96±0.00 | 72.39±5.71 | 69.68±0.00 |
|  |  | F1 | 83.78±0.77 | 84.97±1.02 | 75.70±0.31 | 65.92±5.21 | 82.63±0.15 | 78.45±0.00 | 71.47±0.00 | 81.66±4.12 | 79.85±0.00 |
|  |  | AUC | 64.41±1.82 | 76.12±3.50 | 59.59±1.27 | 61.61±4.45 | 80.00±0.11 | 57.36±0.00 | 64.54±0.00 | 64.17±5.70 | 62.00±0.00 |
|  | Ⅴ | ACC | 65.82±1.87 | 70.04±0.54 | 73.75±0.57 | 62.09±3.39 | 72.69±0.00 | 59.70±0.00 | 58.19±0.00 | 64.60±2.94 | 69.49±0.00 |
|  |  | F1 | 75.80±1.36 | 75.49±0.55 | 84.65±0.45 | 68.39±3.80 | 78.20±0.00 | 66.35±0.00 | 69.67±0.00 | 74.78±2.41 | 78.57±0.00 |
|  |  | AUC | 72.50±2.54 | 78.47±1.05 | 51.99±0.66 | 69.62±2.53 | 79.71±0.03 | 67.43±0.00 | 56.70±0.00 | 59.93±3.60 | 68.23±0.00 |
| **6** | Ⅲ | ACC | 75.33±0.00 | 75.19±1.54 | 73.75±0.09 | 68.69±3.03 | 74.76±0.19 | 75.33±0.00 | 62.52±0.00 | 70.91±2.55 | 73.63±0.00 |
|  |  | F1 | 85.93±0.00 | 85.25±1.00 | 84.86±0.06 | 77.96±2.36 | 80.89±0.20 | 85.93±0.00 | 71.61±0.00 | 79.60±1.23 | 80.28±0.00 |
|  |  | AUC | 64.63±2.58 | 80.49±1.66 | 50.89±0.70 | 63.53±3.40 | 80.40±0.30 | 82.59±0.00 | 70.39±0.00 | 72.02±5.15 | 73.74±0.00 |
|  | Ⅳ | ACC | 73.84±1.18 | 80.57±2.24 | 60.54±2.04 | 76.86±1.47 | 78.37±0.26 | 80.98±0.00 | 64.41±0.00 | 71.97±3.83 | 73.26±0.00 |
|  |  | F1 | 84.02±1.42 | 87.36±1.44 | 72.45±2.04 | 84.56±1.01 | 83.71±0.21 | 86.30±0.00 | 71.92±0.00 | 79.24±1.83 | 79.36±0.00 |
|  |  | AUC | 66.67±1.78 | 87.43±1.68 | 54.16±1.67 | 69.42±2.33 | 86.73±0.10 | 84.91±0.00 | 74.76±0.00 | 71.82±5.65 | 75.09±0.00 |
|  | Ⅴ | ACC | 69.62±2.85 | 73.92±0.53 | 73.52±0.40 | 67.47±2.18 | 76.23±0.18 | 73.26±0.00 | 64.22±0.00 | 74.96±0.99 | 67.04±0.00 |
|  |  | F1 | 77.39±1.54 | 79.36±0.50 | 84.49±0.31 | 73.48±2.10 | 81.54±0.16 | 79.06±0.00 | 74.25±0.00 | 80.66±0.77 | 72.53±0.00 |
|  |  | AUC | 74.61±2.07 | 84.47±1.20 | 52.16±0.12 | 75.44±1.83 | 86.97±0.59 | 78.23±0.00 | 70.76±0.00 | 78.13±2.18 | 77.85±0.00 |
| **7** | Ⅲ | ACC | 75.33±0.00 | 75.05±1.75 | 73.36±0.10 | 70.53±9.15 | 74.73±0.80 | 75.33±0.00 | 63.84±0.00 | 73.69±0.65 | 71.94±0.00 |
|  |  | F1 | 85.93±0.00 | 85.04±1.14 | 84.51±0.05 | 78.75±7.14 | 80.95±0.66 | 85.93±0.00 | 73.48±0.00 | 84.38±0.46 | 81.54±0.00 |
|  |  | AUC | 66.09±3.47 | 80.89±2.08 | 50.16±0.73 | 67.64±9.74 | 80.66±1.09 | 82.59±0.00 | 63.77±0.00 | 53.13±2.42 | 76.32±0.00 |
|  | Ⅳ | ACC | 74.98±0.75 | 81.53±1.64 | 61.21±1.55 | 66.32±1.54 | 78.79±0.36 | 77.59±0.00 | 66.29±0.00 | 70.12±0.73 | 73.07±0.00 |
|  |  | F1 | 84.82±1.44 | 88.20±1.03 | 73.78±1.65 | 77.44±1.25 | 84.03±0.28 | 83.68±0.00 | 74.68±0.00 | 81.50±0.38 | 81.06±0.00 |
|  |  | AUC | 66.33±5.28 | 88.30±1.25 | 49.26±0.84 | 54.49±1.74 | 87.90±0.26 | 85.56±0.00 | 73.18±0.00 | 65.69±8.77 | 76.63±0.00 |
|  | Ⅴ | ACC | 73.28±3.28 | 73.59±0.62 | 74.80±0.17 | 69.10±3.03 | 76.06±0.07 | 74.95±0.00 | 63.28±0.00 | 75.01±2.41 | 73.82±0.00 |
|  |  | F1 | 82.54±3.72 | 79.05±0.62 | 85.32±0.14 | 74.51±3.15 | 81.38±0.06 | 80.35±0.00 | 74.24±0.00 | 81.90±1.39 | 79.88±0.00 |
|  |  | AUC | 75.90±3.32 | 84.16±1.50 | 53.98±0.14 | 78.49±2.08 | 86.72±0.24 | 85.20±0.00 | 60.88±0.00 | 74.83±5.21 | 82.22±0.00 |

**Table S13. The evaluation metrics of the DL models on test set in Group VI and VII (mean ± SD, %).*** "Exp" means the experiment no. in each group.

| **Group.** | **Exp.** | **ACC** | **F1** | **AUC** |
| --- | --- | --- | --- | --- |
| Ⅵ | 8 | 86.45±2.30 | 87.81±2.43 | 92.85±1.82 |
|  | 9 | 78.40±6.21 | 76.91±8.42 | 92.46±1.77 |
|  | 10 | 86.66±3.35 | 87.52±3.64 | 93.16±1.59 |
|  | 11 | 81.46±6.08 | 80.62±7.94 | 94.29±1.06 |
|  | 12 | 87.14±1.93 | 87.93±2.15 | 94.69±0.89 |
|  | 13 | 88.05±1.78 | 88.68±1.98 | 94.87±0.62 |
|  | 14 | 84.56±4.42 | 84.57±5.31 | 94.92±0.92 |
| Ⅶ | 8 | 85.22±2.41 | 87.07±2.52 | 92.74±1.71 |
|  | 9 | 68.39±6.23 | 64.33±9.48 | 91.37±1.46 |
|  | 10 | 70.80±7.21 | 67.88±10.51 | 91.37±2.73 |
|  | 11 | 84.39±2.62 | 85.52±2.93 | 93.68±1.43 |
|  | 12 | 84.77±2.88 | 86.12±3.15 | 93.59±1.86 |
|  | 13 | 83.62±3.54 | 84.44±3.95 | 93.89±1.43 |
|  | 14 | 82.13±3.54 | 82.83±4.08 | 93.92±1.40 |

**Table S14. The evaluation metrics of the DL models on test set in Group VIII - X (mean ± SD, %).** * Exp means the experiment no. in each group.

| **Group.** | **Exp.** | **ACC** | **F1** | **AUC** |
| --- | --- | --- | --- | --- |
| Ⅷ | 8 | 71.22±5.71 | 79.37±4.79 | 76.16±6.39 |
|  | 9 | 73.11±4.65 | 80.24±4.70 | 80.12±3.69 |
|  | 10 | 71.46±2.16 | 77.90±1.45 | 74.98±2.41 |
|  | 11 | 69.15±6.19 | 75.19±6.42 | 80.17±3.05 |
|  | 12 | 72.78±3.12 | 80.02±2.00 | 77.64±3.14 |
|  | 13 | 64.98±8.11 | 69.91±9.61 | 80.78±3.72 |
|  | 14 | 67.28±5.77 | 72.97±6.37 | 80.27±3.15 |
| Ⅸ | 8 | 64.99±8.57 | 70.73±9.32 | 78.08±6.73 |
|  | 9 | 68.04±5.58 | 75.10±6.00 | 76.36±3.58 |
|  | 10 | 74.80±2.40 | 80.80±1.96 | 83.70±3.43 |
|  | 11 | 66.26±7.17 | 72.40±7.98 | 77.77±5.09 |
|  | 12 | 75.91±2.99 | 81.68±2.73 | 84.32±3.17 |
|  | 13 | 76.98±3.84 | 83.23±3.35 | 84.00±2.96 |
|  | 14 | 78.30±3.37 | 84.76±2.87 | 83.92±3.45 |
| Ⅹ | 8 | 74.88±2.06 | 81.46±1.31 | 79.72±2.10 |
|  | 9 | 75.29±1.48 | 81.13±1.10 | 81.59±1.86 |
|  | 10 | 74.27±0.65 | 79.95±0.46 | 77.78±1.66 |
|  | 11 | 76.90±1.29 | 82.45±1.18 | 83.09±1.37 |
|  | 12 | 75.01±1.05 | 80.81±0.74 | 78.47±1.76 |
|  | 13 | 75.19±1.01 | 80.81±0.87 | 81.18±2.04 |
|  | 14 | 76.24±1.21 | 81.75±1.05 | 81.82±1.91 |

**Table S15. The evaluation metrics of the DL models on test set in Group XI - XV (mean ± SD, %).** * Exp means the experiment no. in each group.

| **Group.** | **Exp.** | **ACC** | **F1** | **AUC** |
| --- | --- | --- | --- | --- |
| Ⅺ | 15 | 90.40±0.94 | 91.47±0.93 | 95.20±0.43 |
| Ⅻ | 15 | 88.48±0.88 | 89.92±0.94 | 93.21±0.54 |
| XIII | 15 | 69.99±3.13 | 78.87±1.66 | 77.70±1.70 |
| XIV | 15 | 74.02±3.08 | 81.34±1.93 | 79.47±2.07 |
| XV | 15 | 72.99±1.25 | 79.10±0.69 | 76.47±1.19 |


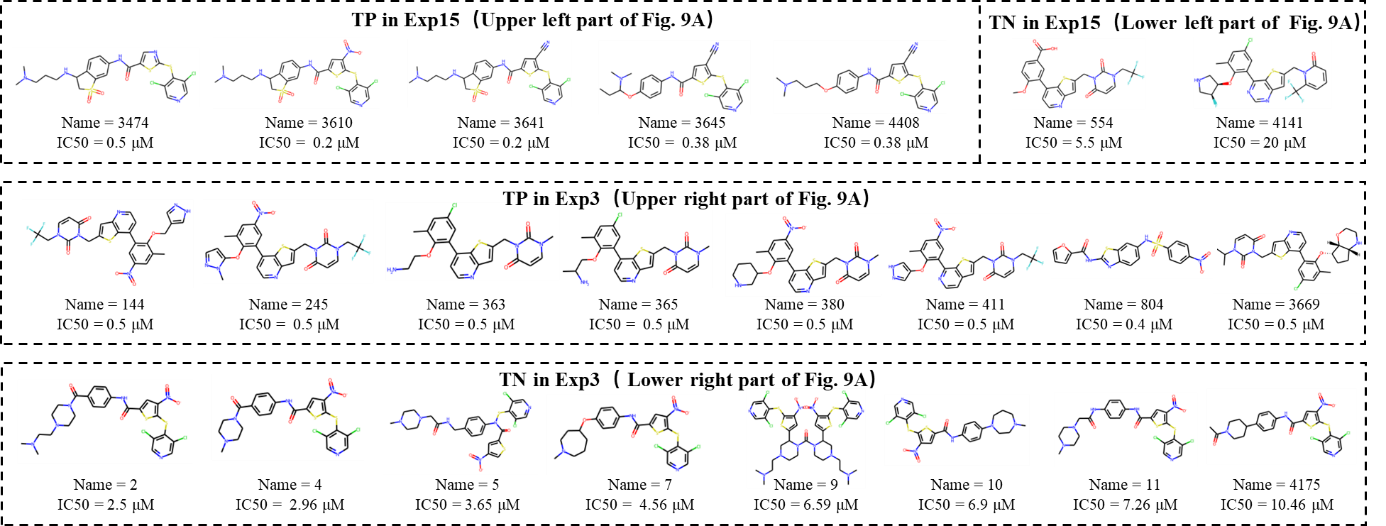


**Fig. S4**. The classifier differences highlighted by Venn diagrams in Fig. 9A


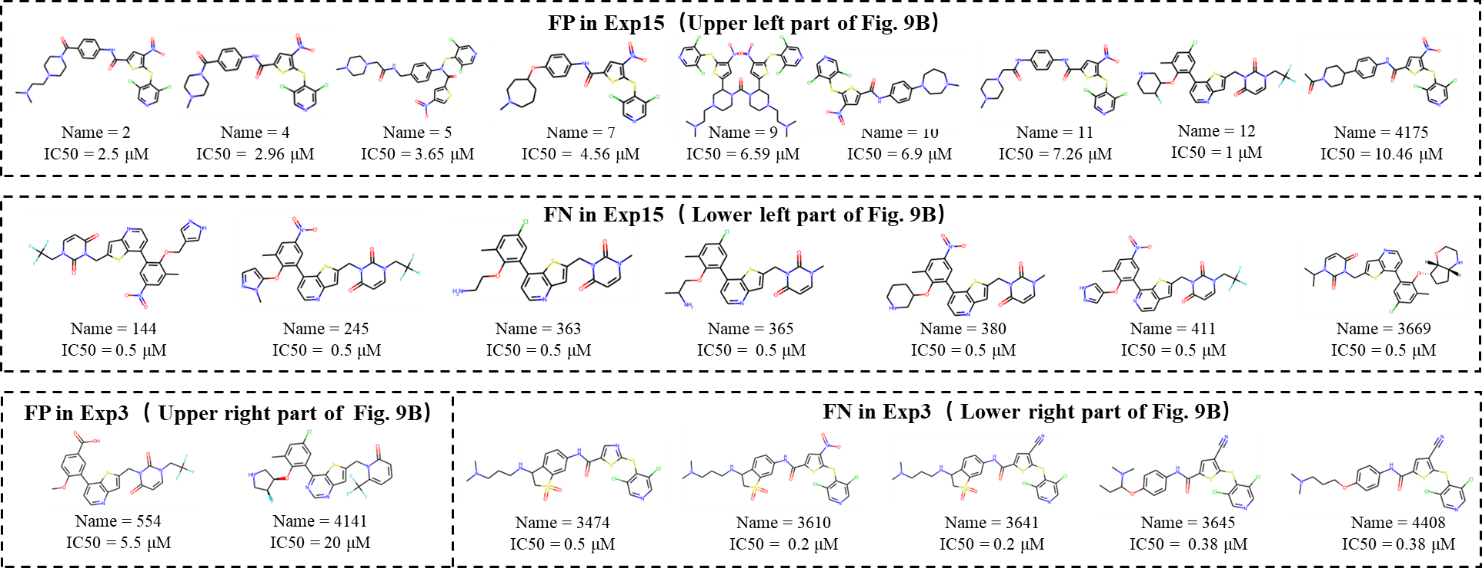


**Fig. S5** The classifier difference highlighted by Venn diagrams in Fig. 9B

**REFERENCES**

1. Andrew Ng, Clustering with the K-Means Algorithm, Machine Learning, 2012

2. Kodinariya, Trupti M., and Prashant R. Makwana. "Review on determining number of Cluster in K-Means Clustering." International Journal 1.6 (2013): 90-95.

3. Shi C, Wei B, Wei S, et al. A quantitative discriminant method of elbow point for the optimal number of clusters in clustering algorithm. Eurasip Journal on Wireless Communications and Networking 2021; 2021:

4. Yang X, Wang Y, Byrne R, et al (2019) Concepts of Artificial Intelligence for Computer-Assisted Drug Discovery. Chem Rev 119:10520–10594.

5. Ezzat, A., Wu, M., Li, X. L., & Kwoh, C. K. (2017). Drug-target interaction prediction using ensemble learning and dimensionality reduction. Methods, 129, 81-88.
